# Identification, synthesis and biological activity of alkyl-guanidine oligomers as potent antibacterial agents

**DOI:** 10.1038/s41598-017-08749-6

**Published:** 2017-08-15

**Authors:** C. Zamperini, G. Maccari, D. Deodato, C. Pasero, I. D’Agostino, F. Orofino, F. De Luca, E. Dreassi, J. D. Docquier, M. Botta

**Affiliations:** 1 Department of Biotechnology, Chemistry and Pharmacy, University of Siena, I-53100 Siena, Italy; 2 Lead Discovery Siena s.r.l., Via Vittorio Alfieri 31, I-53019 Castelnuovo, Berardenga Italy; 3 Department of Medical Biotechnology, University of Siena, I-53100 Siena, Italy; 4 Sbarro Institute for Cancer Research and Molecular Medicine, Temple University, BioLife Science Building, Suite 333, 1900 North 12th Street, Philadelphia, Pennsylvania 19122 United States of America

## Abstract

In the last two decades, the repertoire of clinically effective antibacterials is shrinking due to the rapidly increasing of multi-drug-resistant pathogenic bacteria. New chemical classes with innovative mode of action are required to prevent a return to the pre-antibiotic era. We have recently reported the identification of a series of linear guanidine derivatives and their antibacterial properties. A batch of a promising candidate for optimization studies (compound **1**) turned out to be a mixture containing two unknown species with a better biological activity than the pure compound. This serendipitous discovery led us to investigate the chemical nature of the unknown components of the mixture. Through MS analysis coupled with design and synthesis we found that the components were spontaneously generated oligomers of the original compound. Preliminary biological evaluations eventually confirmed the broad-spectrum antibacterial activity of this new family of molecules. Interestingly the symmetric dimeric derivative (**2**) exhibited the best profile and it was selected as lead compound for further studies.

## Introduction

Bacterial resistance to antibiotics currently represents one of the most relevant threat to global public health^1^ and is responsible for 700,000 deaths every year worldwide^2^. Moreover, the development of new antibiotics, especially active against Gram-negative pathogens, significantly decreased in the last two decades^3–8^. Several factors contributing to the present paucity of new antibacterial were evoked, including poor economic returns^9^, limited throughput of increasingly challenging drug discovery programmes^10^ and the complexity of the regulatory process to obtain drug approval^6, 11–13^. Consequently, few new antibacterials with novel mechanisms of action have been approved by FDA during the last 20 years, for example linezolid in 2000^14^, daptomycin in 2003^15^ and ceftaroline fosamil in 2010^16^, all being active only against Gram-positive pathogens^17–20^. The rapid evolution of relevant Gram-negative pathogens (*Klebsiella pneumoniae*, *Pseudomonas aeruginosa* and *Acinetobacter baumannii*) exhibiting extensively drug- and pandrug-resistance phenotypes ultimately limits the efficacy of most available antibiotics, including the life-saving carbapenems and colistin^4, 21–27^. In the frame of this unprecedented “antibiotic crisis”^28^, and to avoid a return to the pre-antibiotic era, the discovery and development of new active antibacterials, belonging to novel chemical classes, is urgently needed^1, 29^.

It is known that many compounds which show a significant antibacterial activity are characterized by the presence of one or more guanidine functions in their structure^30–32^. At physiological pH, the guanidine moiety carries a positive charge^33^ and the supposed mechanism of action involves an electrostatic interaction between the negatively charged bacterial cell surface and the positively charged compounds, potentially resulting in disruption of the bacterial membrane integrity. The increased membrane permeability thus leads to lysis and cell death^30^. Amphiphilic molecules with a hydrophobic surface and a net positive charge are called CADs or CAPs (Cationic Amphiphilic Drugs or Peptides) and act through the above described interaction, inducing an alteration of phospholipids storage^34–36^.

In our previous work, we reported the synthesis and the biological evaluation of a series of linear alkyl-biguanylated compounds showing a subnanomolar affinity (*K*_*i*_ ranging from 0.08 to 3.00 nM) as competitive inhibitors of Maize polyamine oxidase (PAO). The selective binding with this enzyme plays a crucial role in the inhibition of cell proliferation, in particular in tumor cell lines^37^.

**Figure 1:**
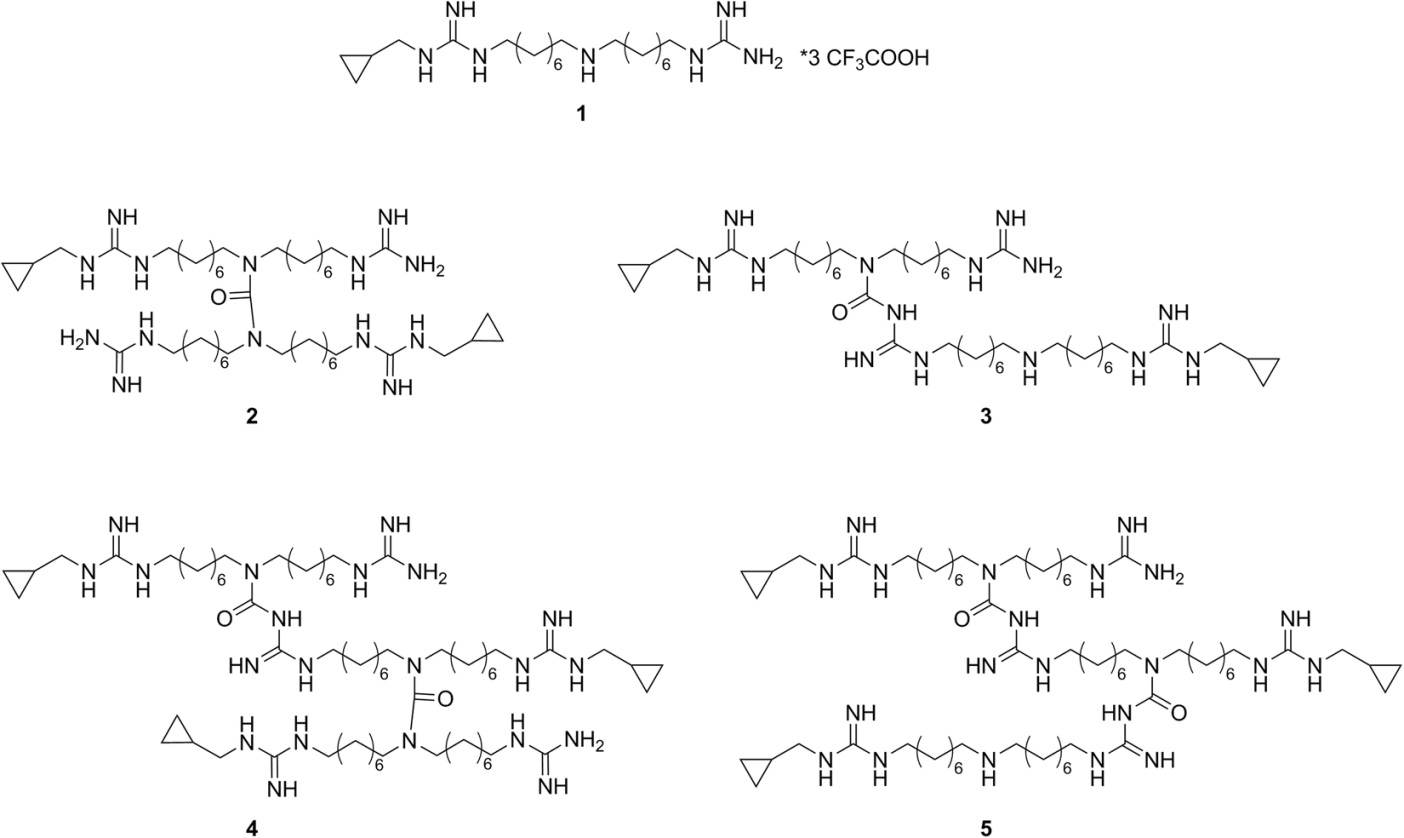
Structures of compound **1** and of the dimers (symmetric and asymmetric: **2** and **3** respectively) and trimers (symmetric and asymmetric: **4** and **5** respectively) studied in this work.

Considering this important correlation between the guanidine moiety and the antimicrobial properties, we decided to evaluate the antibacterial profile of some of the above mentioned anti-PAO derivatives. The activity of some selected molecules was tested on a panel of different bacteria, including representatives of both Gram-positive and Gram-negative organisms and clinical isolates, allowing the identification of compound **1** (Fig. 1) as promising candidate for further development. Interestingly, it exhibited a potent antibacterial activity on Gram-positive strains with MIC values ranging from 0.12 to 4 µg/mL and a remarkable activity on multi-drug resistant clinical isolates of *E. cloacae* and *A. baumannii* (MIC values of 2 and 4 µg/mL respectively)^38^.

In order to perform further analysis on compound **1**, a new synthetic strategy (“Chemistry”) has been set up to overcome the withdrawal from the market of some commercial starting materials. The biological assays conducted on all the newly synthesized batches of compound **1** surprisingly showed a significantly lower antibacterial activity, when compared to that of the original batch of compound **1** ^38^. In the light of these results, we turned our attention to this first batch to understand the reason of its higher activity. By means of analytical procedures (HPLC-MS) carried out on this sample, it emerged that it actually consisted of a multicomponent mixture including three different chemical identities. Initial attempts to separate the main components of the mixture with previously optimized HPLC-MS protocols did not allow a complete separation of all species but successfully separate only compound **1** from the other analytes that were collected together and tested, revealing to have the better activity profile. Hence, we investigated about their chemical nature and through accurate mass measurements and MS^n^ experiments, we hypothesized that they could be dimer and trimer of compound **1**. We designed two possible isomers for each oligomer, as reported in Fig. 1 ^39^. At least, dimers (**2** and **3**) and trimers (**4** and **5**) have been synthesized and tested separately to identify the real responsible for the high antibacterial activity.

## Results and Discussion

### HPLC and MS analysis

**Figure 2:**
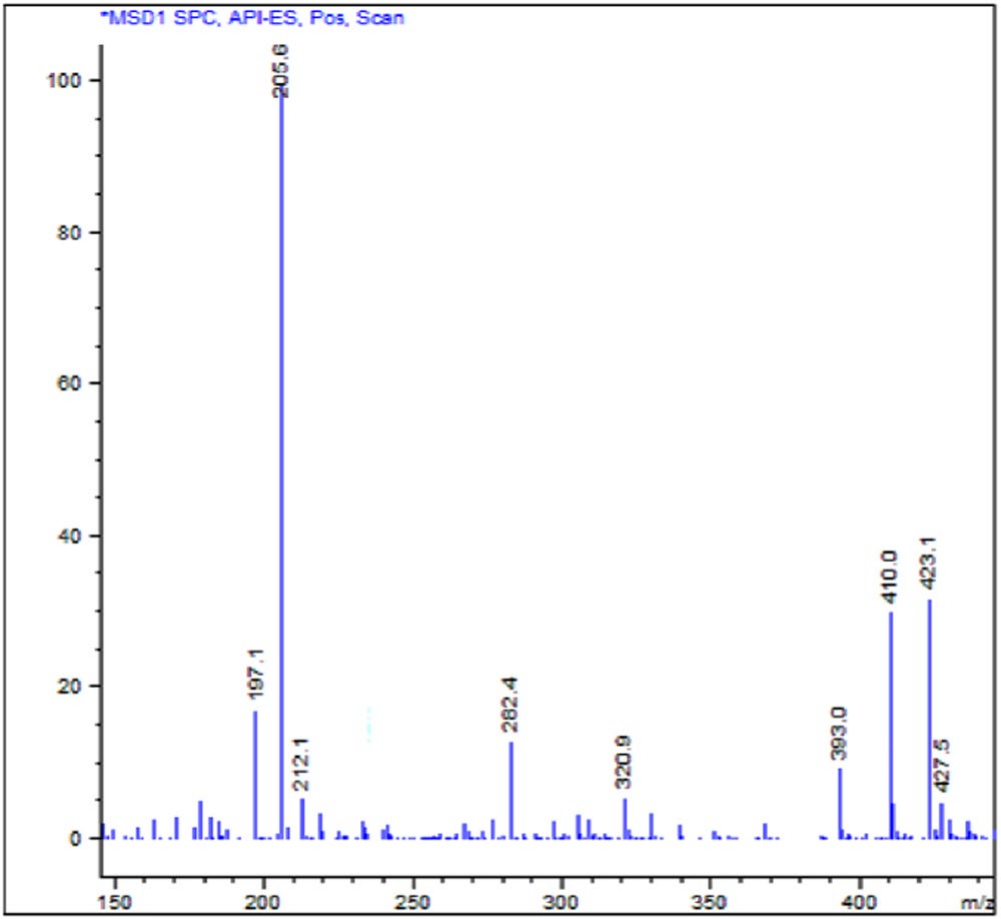
Mass spectrum obtained by direct injection of a sample of the mixture. Conditions described in “Methods - *General chemistry*”.

Fig. 2 shows the mass spectrum obtained by a direct injection of a sample of the original batch of compound **1**. Signals detected were attributable to molecular structures heavier than compound **1**. Further in-depth studies allowed the assignment of each MS signal to multiple charged ions of oligomeric derivatives, characterized as shown in Table 1.

**Table 1:** Chromatographic and mass spectral data of compounds present in the analyzed mixture.

Eluate	Compound	Mean t_R_ ± SD^*a*^ (min)	Principal ions (*m/z*)^*b*^			
			[M + H]^+^	[M + 2 H]^2+^	[M+3 H]^3+^	[M+4 H]^4+^
A	Monomer (**1**)	12.44 ± 0.027	410.1	205.5	137.4	—
B	Dimer	13.48 ± 0.032	845.8	423.1	282.5	212.1
C	Trimer	13.88 ± 0.025	1281.1	641.1	427.5	320.9

^*a*^Chromatographic conditions reported in “Experimental section - *Chromatographic separation*”.

^*b*^Ions detected with Agilent 1100 series MSD single-quadrupole instrument with fragmentation voltage 30 mV, reported in “Experimental section - *Preliminary characterization studies*”.

**Figure 3:**
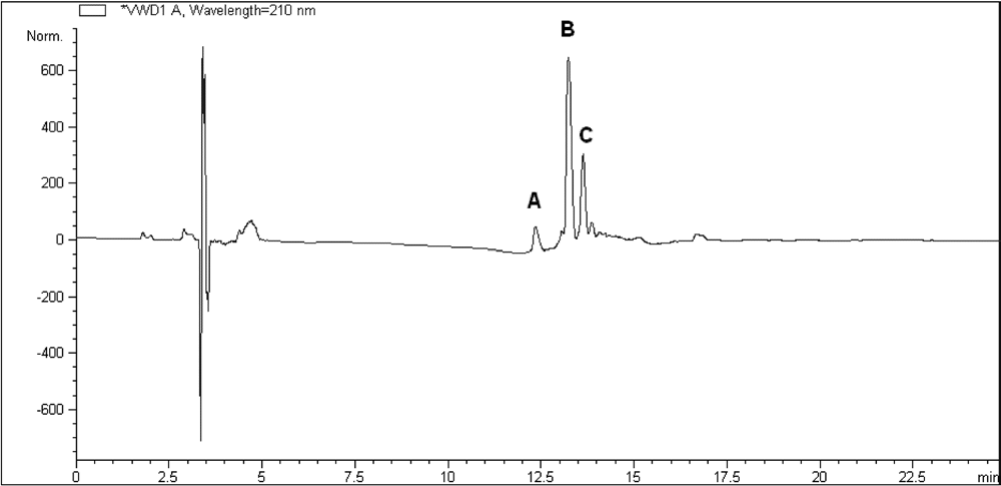
Chromatographic profile obtained after blank substraction of a sample of the first batch (10 mg/mL) by LC–MS:**A** (t_R_ = 12.40 min) monomer (**1**); **B** (t_R_ = 13.34 min) = dimer; **C** (t_R_ = 13.74 min) = trimer. Chromatographic method and conditions are reported in “Methods - *Chromatographic separation”*.

Attempts of identification and separation of the main components of the mixture were performed through LC–MS method, using a C18 column with a linear gradient elution, which gave us the best results. The chromatographic profile obtained is reported in Fig. 3.

The HPLC trace shows three main components, as the UV signals A, B and C, corresponding respectively to compound **1** and two other larger species, with *m/z* values approximatively twice and three times higher than **1** (see Supplementary Fig. S1). The eluate A was isolated and identified as compound **1**, while the analytes B and C were collected together in a single fraction because of their close retention times, not giving us the information necessary to identify them. The eluted fractions were tested separately, revealing in the case of compound **1** (eluate A) the same moderate antibacterial activity of its freshly synthesized batches. On the other side, the fraction containing the eluates B and C showed MIC values comparable to that of the original batch, confirming that its good antibacterial profile was due to the components contained in this latter fraction. Hence, we decided to investigate about their chemical nature. Preliminary fragmentation studies obtained by changing the fragmentor voltage were performed on a sample of the original batch (see Supplementary Figs S2 and S3) and showed, at higher fragmentation energy, the presence of fragments of compound **1** in all the three chromatographic peaks; this observation demonstrates that the unknown components could be derivatives of compound **1**. At lower fragmentation energy, instead, the double, triple and quadruple charged cations prevailed. Chromatographic and mass data, resumed in Table 1, led us to hypothesize that the unknown mixture components could be a dimer and a trimer, characterized by a carbonyl group as the linker between the monomers (compound **1**).

Although the factors favoring the formation of these derivatives were unclear, we assumed that the generation of this mixture occurred during the storage of the sample in DMSO solution before the biological evaluation, especially considering that the characterization analysis of compound **1**, performed immediately after its synthesis, confirmed its purity and authenticity.

Moreover, in-depth studies were performed to elucidate the chemical formula and the structure of the main components. Through MS (ES+) analysis nowadays it is possible to obtain structurally significant fragment patterns^40^. LTQ-Orbitrap is a LC/MS technique usually used in analysis of unknown or peptidic mixtures because of its very high-resolution and high mass accuracy measurements on molecular ions^41^. One of its recent approaches is reported as the structural identification of drug metabolites^42, 43^. In this study accurate mass measurements and empirical formula calculations for the molecular ions were conducted using LTQ-Orbitrap XL mass spectrometer **(**Table 2). The ring and double bond (RDB) values and the difference between the theoretical (dimeric and trimeric) and experimental *m/z* for product ions (Delta) supported our hypothesis.

**Table 2:** Accurate mass data.

Eluate	Compound	Accurate mass determination of pseudomolecular ions^*a*^			
		Molecular Formula	[M + H]^+^ (*m/z*)	Delta (ppm)	RDB
A	Monomer (**1**)	C_22_H_48_N_7_	410.39545	−2.733	2.5
B	Dimer	C_45_H_93_O_1_N_14_	845.76379	−1.585	6.5
C	Trimer	C_68_H_138_O_2_N_21_	1281.13330	−0.305	10.5

^*a*^Accurate mass measurements and chemical formulas calculation were found through LTQ-Orbitrap XL and proposed chemical formulas, *m/z* values, RDB and Delta values were obtained from the software Xcalibur (Thermo Scientific, Bremen, Germany), as reported in “Experimental section *- Accurate mass and fragmentation studies*”.

On the basis of the detected properties, we designed two possible structural isomers for each oligomer: a symmetric and an asymmetric one, as reported in Fig. 1. We refer to asymmetric structure (compounds **3** and **5**) when the connection between the monomers involves the central amine of one monomer and the guanidine group of the other, generating an amidinourea moiety. On the other side, the symmetric structure (compounds **2** and **4**) is characterized by a urea function, involving both the central amines of the two monomers.

**Figure 4:**
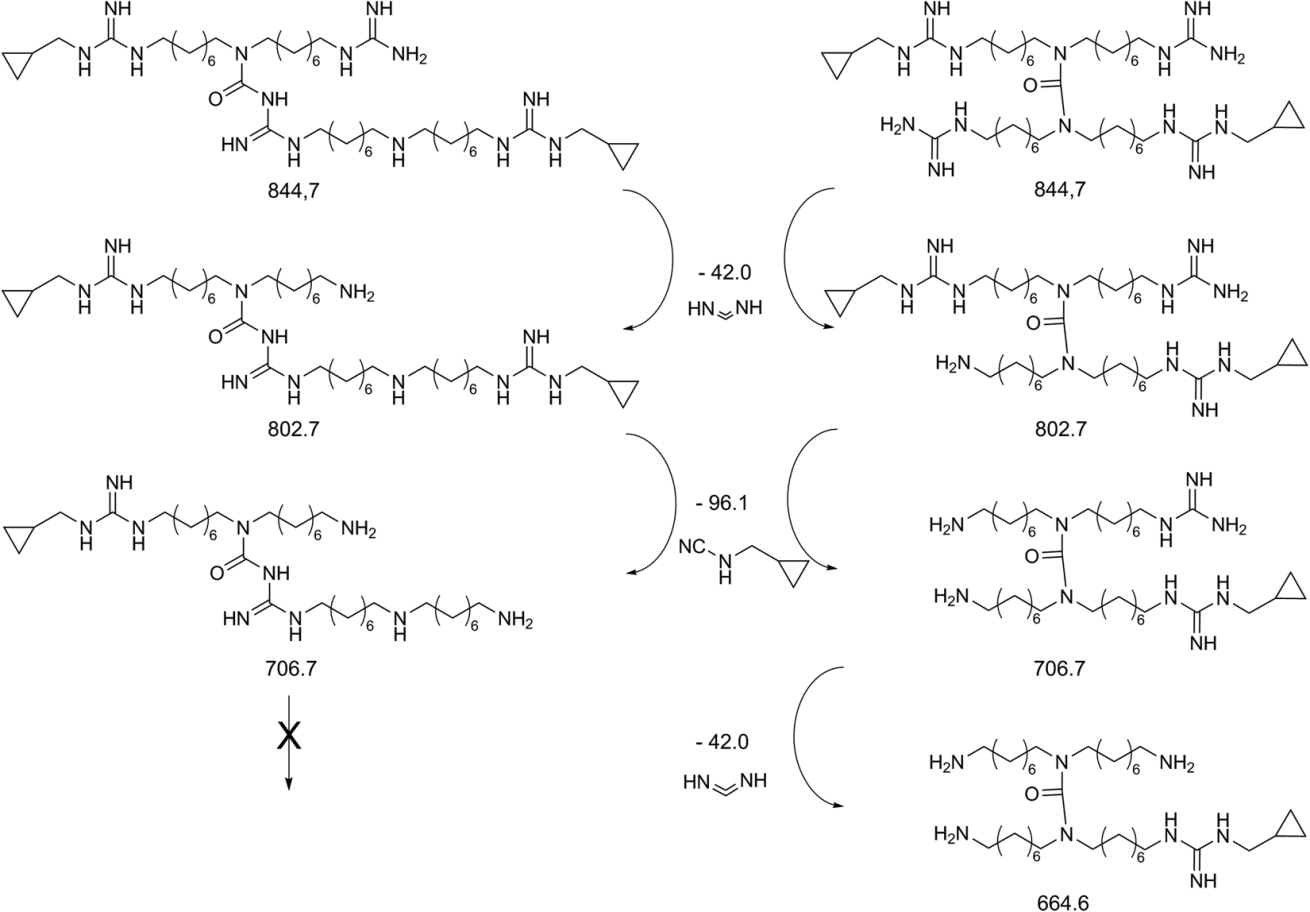
Proposed fragmentation pattern for the structures of dimeric moiety and their calculated exact mass. Spectral details are reported in “Supplementary Information”.

To establish which was the actual structure of dimer and trimer between the two hypothesized isomers, the mixture was analyzed by per infusion MS^n^ technique, using an ion trap coupled with the Orbitrap mass analyzer, that allows fast, sensitive and reliable detection and identification of small molecules regardless of relative ion abundance analytes^44, 45^. The MS^2^ and MS^3^ spectra obtained from the precursor ion 845.7 *m/z* showed the formation of several product ions, in particular we detected 803.9 and 707.8 *m/z*, derived from the loss of methanediimine and *N*-(cyclopropylmethyl)-cyanamide fragments respectively, which are characteristic of both symmetric and asymmetric isomers (see Supplementary Figs S4 and S5). The detection in MS^4^ experiments of the signal at 665.8 *m/z*, due to the loss of another methanediimine fragment (see Supplementary Fig. S6), confirmed the symmetric structure of the dimer (**2**) and/or the trimer (**4**), since this fragmentation is not possible for the asymmetric isomers (**3** and **5**), as reported in Fig. 4.

From the isolation of the trimer signal at 1281.1 *m/z*, MS^2^ spectrum showed 845.8 and 410.5 *m/z* as the main signals, corresponding to dimer and monomer respectively (see Supplementary Fig. S7).

This per infusion MS^n^ technology led us to observe the presence of a symmetric moiety that could belong to the dimer or the trimer. Unfortunately, this moiety was not assignable to one of the two compounds since the retention times were too close to perform this kind of experiment during the chromatographic run. For this reason, to confirm the structure of the dimer and the trimer present in the original mixture we turned to the synthesis of all the possible isomers shown in Fig. 1.

Compounds **2**–**5** obtained by the synthesis^39^ were analyzed through HPLC-MS to compare their retention time with the ones of the initial mixture. The chromatograms showed a perfect correspondence between the two symmetric isomers (**2** and **4**) and the eluates B and C of the mixture. Accurate mass experiments of compounds **2** and **4** have been conducted, revealing that these two compounds were the mixture predominant components.

Moreover, a quantitative analysis of the fraction containing eluates B and C was performed with the same separation method above mentioned and it revealed that the B/C ratio, corresponding to the molar ratio of compound **2**/compound **4**, was 7/3. This molar ratio was extrapolated from appropriate standard calibration curves of compounds **2** and **4** obtained through HPLC-UV/MS signals.

### Chemistry

**Figure 5:**
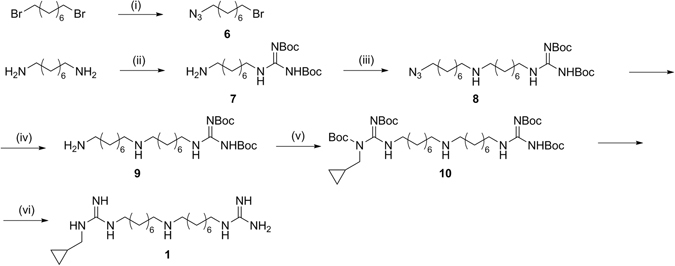
Synthesis of monomer (**1**). *Reagents and conditions:* (i) NaN_3_, DMF, 50 °C, 16 h; (ii) 1,3-Bis(tert-butoxycarbonyl)-2-methyl-2-thiopseudourea, DIPEA, CH_3_CN/CH_3_OH, 40 °C, 16 h; (iii) CsOH·H_2_O, molecular sieves, **6**, dry DMF, r.t., 24 h; (iv) H_2_, Pd/C, *i*-PrOH, r.t., 4 h; (v) *N,N*′-Di-Boc-*N*-methylcyclopropyl-pyrazole-1-carboxamidine, DIPEA, THF, 16 h, r.t.; (vi) TFA 10%, dry DCM, r.t., 7 h.

The synthetic procedure for the preparation of compound **1** reported in the literature^37^ is not accessible since the starting material 1,17-diamino-9-azaheptadecane is no longer commercially available. Thus, we reported in Fig. 5 a new strategy which begins with the guanylation of 1,8-diaminooctane, using 1,3-Bis(tert-butoxycarbonyl)-2-methyl-2-thiopseudourea, furnishing **7**. The monoguanylated derivative was reacted with **6**, previously obtained from 1,8-dibromooctane via nucleophilic substitution. The azido group of the resulting **8** was reduced and guanylated with *N,N*′-Di-Boc-*N*-methylcyclopropyl-pyrazole-1-carboxamidine, giving **10**. Final compound **1** was obtained as trifluoroacetate salt after deprotection of all the Boc protecting groups with TFA in DCM.

The guanylating agent, *N,N*′-Di-Boc-*N*-methylcyclopropyl-pyrazole-1-carboxamidine, have been obtained through Mitsunobu reaction between cyclopropanemethanol and di-Boc-pyrazole-1-carboximidamide, as reported in our previous work^38, 39^.

**Figure 6:**
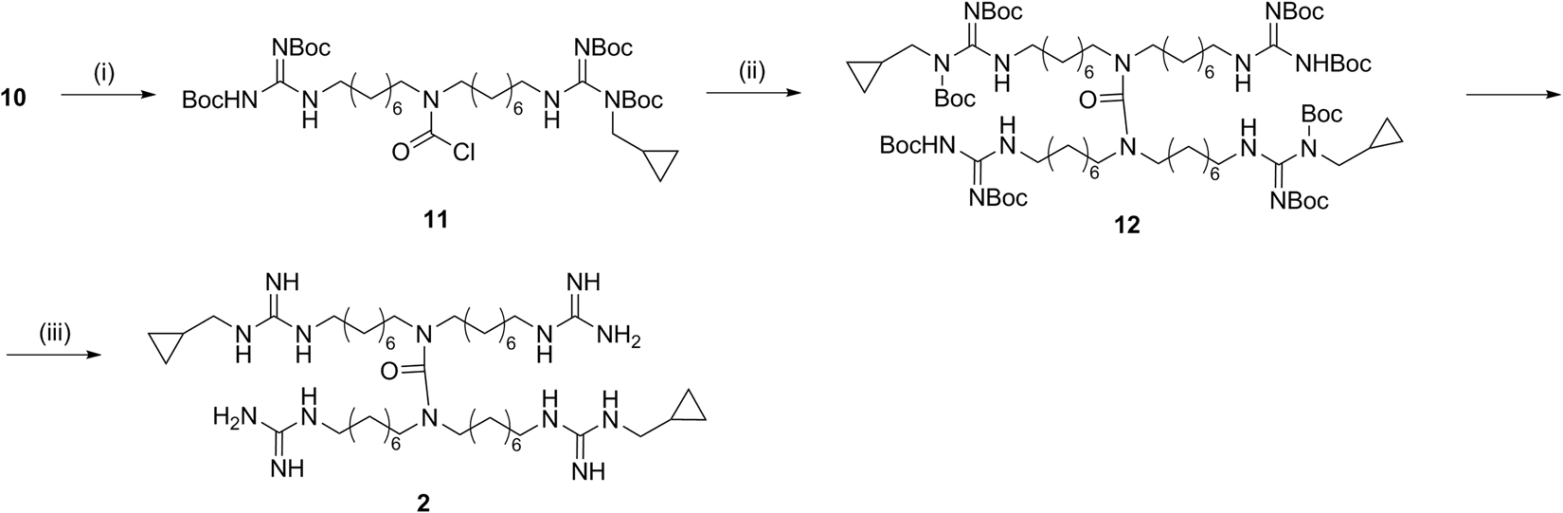
Synthesis of symmetric dimer (**2**). *Reagents and conditions*: (i) Triphosgene, DIPEA, dry THF, 0 °C to r.t., 10 min; (ii) **10**, DIPEA, NaI, dry DCM, 40 °C, 48 h; (iii) TFA 10%, dry DCM, r.t., 7 h.

As reported in Fig. 6, to synthesize the symmetric dimer **2**, compound **10** was reacted with its carbamoyl derivative (**11)** affording the urea function. At the end the dimer **12** was deprotected under acidic condition, furnishing the trifluoroacetate salt of the final product (**2**).

**Figure 7:**
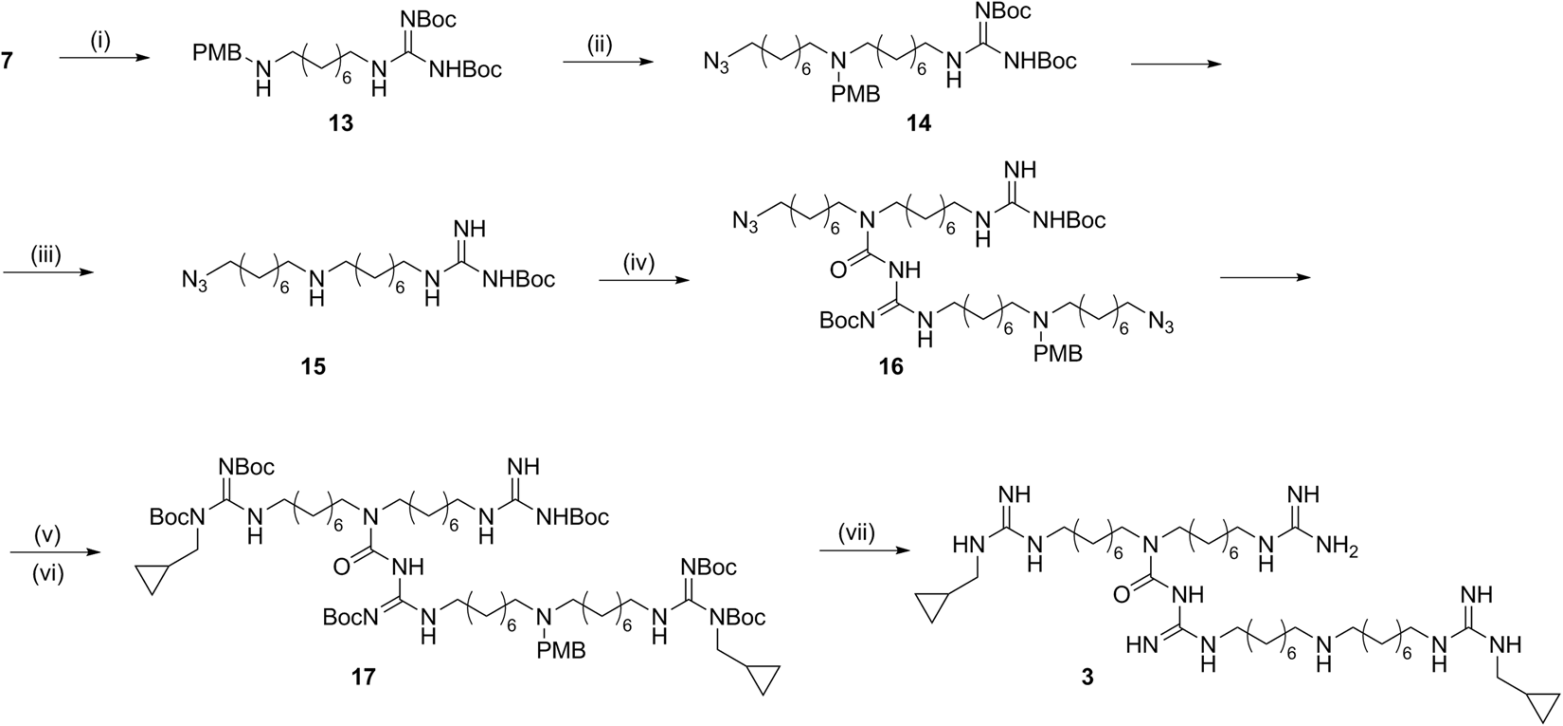
Synthesis of asymmetric dimer (**3**). *Reagents and conditions:* (i) 1) *p*-Anisaldehyde, CH_3_OH, r.t., 3 h; 2) NaBH_4_, 0 °C, 1 h; (ii) **6**, NaI, DMF, r.t., 48 h; (iii) CAN, *t-*BuOH/CH_3_OH, 55 °C, 5 h; (iv) **14**, TEA, dry THF, ref., 2 h; (v) H_2_, Pd/C, *i-*PrOH, r.t., 16 h; (vi) *N,N*′-Di-Boc-*N*-methylcyclopropyl-pyrazole-1-carboxamidine, DIPEA, THF, r.t., 16 h; (vii) TFA 10%, dry DCM, r.t., 7 h.

The preparation of the asymmetric dimer **3** (Fig. 7) was more challenging, because required an orthogonal reaction between the central amine of a first monomer and the carbonyl group of a Boc of a second one^46, 47^. In order to promote the cross-reaction over oligomerization and self-cyclization, we designed and synthesized two different monomers (**14** and **15**) in such a way that they would react in an orthogonal fashion. Thus, the secondary amine of **14** was protected with a p-methoxybenzyl (PMB) group and, since the coupling step requires a di-Boc-guanidine moiety to be successful^46, 47^ the guanidine function of **15** was inactivated as mono-Boc-protected. The two building blocks were synthesized from compound **7** that was first protected with the PMB group and then reacted with 1-azido-8-bromooctane **6**, furnishing **14**. The other monomer (**15)** was synthesized from compound **14** through an oxidative deprotection via cerium ammonium nitrate. This reaction led to the simultaneous removal of the PMB and one Boc on the guanidine moiety. **15** was then reacted with compound **14** to give the dimeric compound **16**. Reduction of the azido groups and their following guanylation afforded **17**, that was eventually deprotected to give the asymmetric dimer **3** as trifluoroacetate salt.

**Figure 8:**
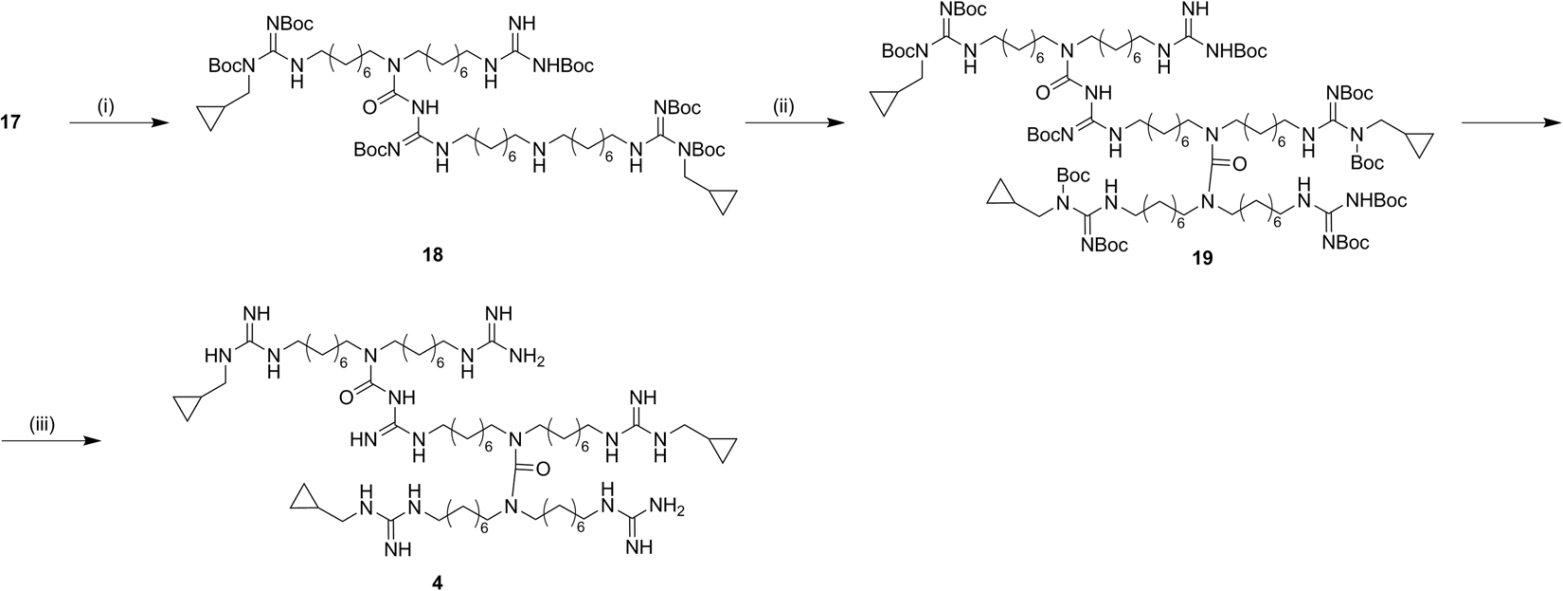
Synthesis of symmetric trimer (**4**). *Reagents and conditions*: (i) CAN,﻿CH_3_CN/H﻿_2_O﻿, r.t., 16 h; (ii) **11**, DIPEA, NaI, dry DCM, ref., 8 h; (iii) TFA 10%, dry DCM, r.t., 7 h.

In the synthesis of trimer **4**, to selectively remove the PMB group from **17**, without removing the Boc protecting groups, the oxidative deprotection via cerium ammonium nitrate was set up with different conditions

and reaction time, allowing the obtainment of **18**. Its free central amine was reacted with carbamoyl derivative **11** to obtain, after acidic deprotection, the trifluoroacetate salt of the symmetrical trimer **4**. (Fig. 8)

**Figure 9:**
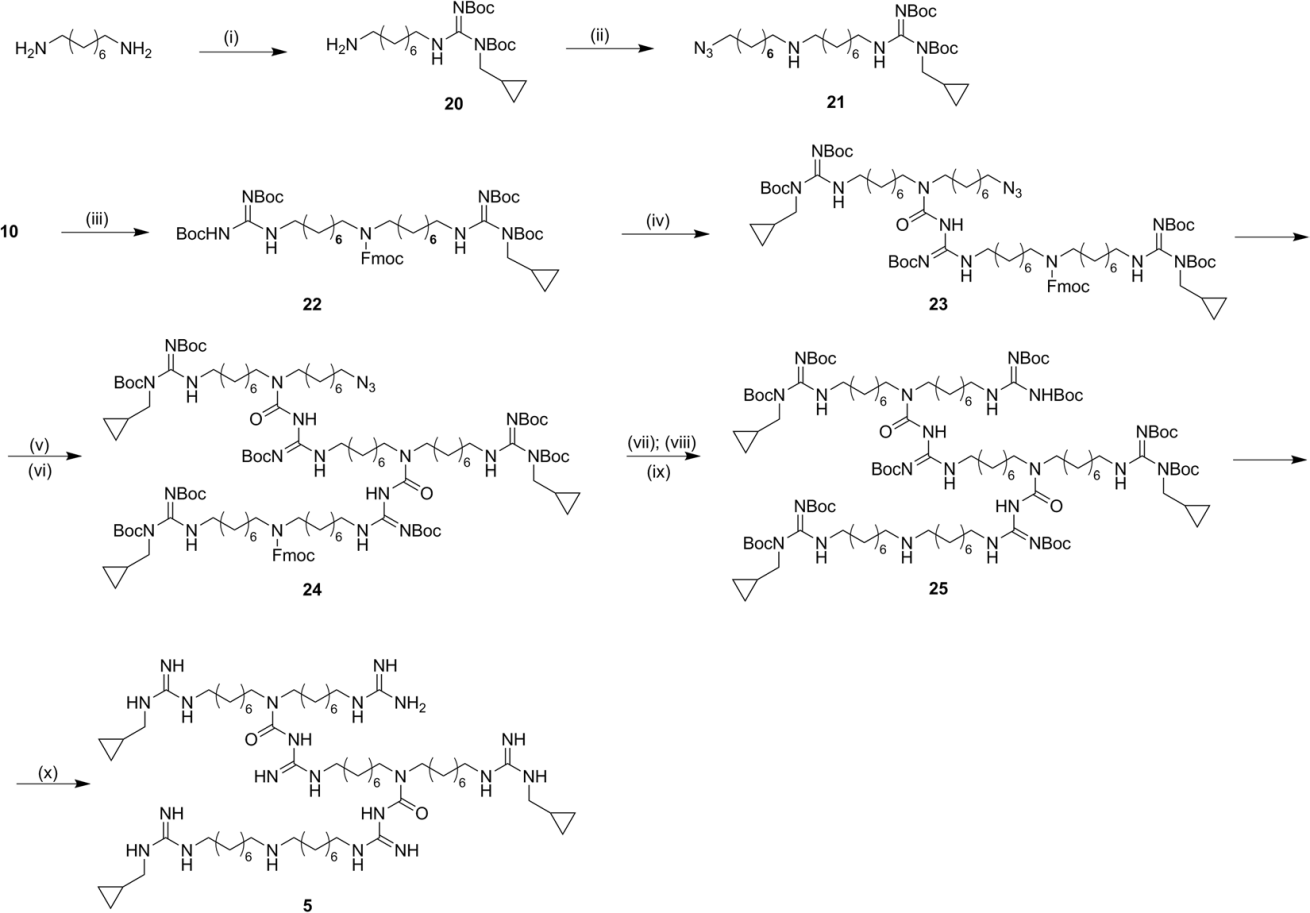
Synthesis of the asymmetric trimer (**5**). *Reagents and condition:* (i) *N,N*′-Di-Boc-*N*-methylcyclopropyl-pyrazole-1-carboxamidine, DIPEA, CH_3_CN/CH_3_OH, 50 °C, 16 h; (ii) CsOH·H_2_O, molecular sieves, **6**, dry DMF, r.t., 24 h; (iii) FmocCl, DIPEA, dry DCM, 0 °C to r.t., 2 h; (iv) **21**, TEA, THF, ref., 10 h; (v) Piperidine 20%, DMF, r.t., 1 h; (vi) **22**, TEA, dry THF, ref., 16 h; (vii) H_2_, Pd/C, *i-*PrOH, r.t., 1.5 h; (viii) *N*,*N*′-Di-Boc-1*H*-pyrazole-1-carboxamidine, DIPEA, THF, r.t.,16 h; (ix) Piperidine 20%, DMF, r.t., 5 h; (x) TFA 10%, dry DCM, r.t., 10 h.

Central amine of monomer **10** was protected with FMOC, affording **22**, and reacted with **21**, which was obtained with the same synthetic procedure described for **8**. The resulting **23** was first deprotected from FMOC and then reacted with **22**, affording **24**. **25** was easily prepared with subsequent reduction, guanylation and FMOC deprotection. Final removal of Boc protecting groups, under acidic condition, gave the asymmetric trimer **5** as trifluoroacetate salt. (Fig. 9)

### Antibacterial activity

The antibacterial activity of eluate A (compound **1** isolated from the mixture), eluates B and C together and compounds **1–5** was investigated and their MIC values determined using a panel of eight organisms representative of both Gram-positive and Gram-negative bacteria. Compound **1**, both the newly synthesized and the one isolated as eluate A, surprisingly showed a lower activity, when compared to that of the original batch published in our previous work^38^(reported as “Initia﻿l mixture” in Table 3), supporting the fact that the observed antibacterial activity was actually due to the presence of the other chemical species. In fact, the fraction containing both eluates B and C exhibited a significant antibacterial activity, demonstrating to be composed by the active molecule(s) of the original batch. All the synthesized oligomers also showed a notable antibacterial activity, especially on Gram-positive organisms but showed a different biological profile according to their isomerism: the symmetric isomers (**2** and **4**) were more active than their asymmetric counterparts (**3** and **5**). The symmetric dimer (**2**) exhibited the most potent activity on all the tested organisms, instead the asymmetric dimer (**3**) apparently lost most of its activity on Gram-negative ones. The symmetric trimer (**4**) was moderately active against all the tested species, while the asymmetric one (**5**) had a good activity on Gram-positive pathogens, in particular *E. faecalis*.

**Table 3:** Minimum Inhibitory Concentration (MIC) of compounds **1**–**5**, eluates A, B and C and control antibiotics (colistin, vancomycin and daptomycin) against representative strains of Gram-positive and Gram-negative bacteria.

Compound	MIC (µg/mL)^*a*^							
	*E. c*.	*K. p*.	*P. a*.	*A. b*.	*S. e*.	*E. f*.	*B. s*.	*S. a*.
Initial mixture^*b*^	0.5	1	8	4	0.5	1	0.5	4
Eluate A (**1**)	64	> 64	64	> 64	64	> 64	64	> 64
Eluates B and C	1	2	16	16	0.5	1	0.5	—
**1** ^*c*^	64	> 64	> 64	> 64	> 64	> 64	64	> 64
**2**	2	2	8	8	1	2	0.5	2
**3**	> 64	> 64	> 64	> 64	8	8	< 0.125	4
**4**	4	4	16	8	4	4	4	8
**5**	8	8	32	32	4	< 0.125	8	8
Colistin	0.5	0.5	0.5	1	—	—	—	—
Vancomycin	—	—	—	—	2	1	0.5	1
Daptomycin	—	—	—	—	0.25	1	1	0.5

-.: not determined.

^*a*^MICs are expressed as median values calculated from experiments performed at least in triplicate.

Tested bacterial strains: *E.c.: E. coli* CCUG^T^; *K.﻿ ﻿p.: K. pneumoniae* ATCC 13833; *P.a.: P. aeruginosa* ATCC 27853; *A.b.: A. baumannii* ATCC 17978; *S.e.: S. epidermidis* ATCC 14990; *E.f.: E. faecalis* ATCC 29212; *B.s.: B. subtilis* ATCC 6633; *S.a.: S. aureus* ATCC 25923.

^*b*^First batch of compound **1** as published^38^ that turned out to be a mixture of eluates A, B and C.

^*c*^Newly synthesized batches of compound **1**.

These antibacterial activity data overall support that the two symmetric oligomers were most likely the active components of the original mixture, thus confirming our hypothesis.

The biological profile of compound **2** which was the most active of the series was further investigated through Minimal Bactericidal Concentration (MBC) assay to distinguish whether it is bactericidal or bacteriostatic. Upon analysis, the MBC values of compound **2** on some bacterial strains were determined and subsequently compared to the corresponding MIC values: compound **2** was found to be bactericidal at the same concentrations to its MICs. As per CLSI standards^48^, a MBC/MIC ratio of 1 to 2 is considered indicative of bactericidal behaviour; while a ratio ≥ 8 is indicative of bacteriostatic activity. Compound **2** showed a strong bactericidal activity with MBC and MIC values found to be identical for all the tested organisms.

Furthermore, the determination of killing curves showed that compound **2** was a relatively fast bactericidal agent, with a reduction of viable microbial load of > 3 log_10_ after only 1 h of exposure to the compound (final concentration, 10 × MIC) (see Supplementary Fig. S10). A further reduction of the viable count was progressively observed (>5 log_10_ after 4 h) and no viable cells could be detected after 24 h. Interestingly, compound **2** did not show any detectable haemolytic activity (up to 50 µg of compound tested in the assay).

## Conclusion

In summary, in-depth MS studies allowed the identification of the composition of a spontaneously generated mixture derived from a batch of compound **1**. We assume it originated after the re-suspension and the storage of the pure compound in DMSO prior to the evaluation of its antibacterial properties, as explained in Supplementary Information. We designed four possible isomers of the main components and synthesized them separately. Biological data have highlighted compound **2** as the actual responsible for the antibacterial activity with MIC values ranging from 1 to 8 µg/mL. It showed a strong bactericidal activity on both Gram-positive and Gram-negative clinically-relevant pathogens, while no haemolytic activity could be detected. Remarkably, this work originated from a serendipitous discovery and contributed to the identification of a new chemical scaffold showing a broad-spectrum antibacterial activity. Compound **2** was chosen as lead compound for further investigations to generate a library of derivatives. These findings represent a significant achievement considering the current need of novel classes of antibacterials to fight resistant bacteria.

## Methods

### General Chemistry

All commercially available chemicals and solvents were used as purchased. DCM was dried over calcium hydride and THF was dried over sodium and benzophenone prior to use. Anhydrous reactions were run under a positive pressure of dry nitrogen. Chromatographic separations were performed on columns packed with silica gel (230–400 mesh, for flash technique). ^1^H NMR and ^13^C NMR were recorded at 400 and 100 MHz respectively on a Bruker AC200F spectrometer and are reported in parts per million (δ scale) and internally referenced to the CDCl_3_ or CD_3_OD signal, respectively at δ 7.24 ppm and 3.31 ppm. Chemical shifts for carbon are reported in parts per million (δ scale) and referenced to the carbon resonances of the solvent (CDCl_3_ at δ 77.00 and CD_3_OD at δ 49.00 ppm). Data are shown as following: chemical shift, multiplicity (s = singlet, d = doublet, t = triplet, m = multiplet and/or multiplet resonances, br = broad), coupling constant (*J*) in Hertz (Hz) and integration. Mass spectra (LC-MS) were acquired using an Agilent 1100 LC-MSD VL system (G1946C) by direct injection with a 0.4 mL/min flow rate using a binary solvent system of 95/5 CH_3_OH/H_2_O. UV detection was monitored at 254 nm. Mass spectra were acquired in positive mode scanning over the mass range 105–1500 *m/z*, using a variable fragmentor voltage of 10–70 mV.

### Determination of the purity

The purity of final products (**1**–**5**) was 95% or higher and it was assessed by HPLC-MS, using an Equivalence 3 C18 column (ACE EQV-8977: 150 × 4.6 mm, 5 μm particle size) at a flow rate of 0.6 mL/min with a linear gradient elution from 100/0 to 50/50 v/v CH_3_CN (formic acid 0.1% v/v)/H_2_O (formic acid 0.1% v/v) for 23 min. UV detection was monitored at 210 nm. Mass spectra were acquired in positive mode scanning over the mass range 105–1500 *m/z*, using a fragmentor voltage of 70 mV.

### Interference compound prediction

The behaviour of all the final compounds (**1**–**5**) as PAINS was predicted using the web-server FAFDrugs^3^. Through its tool Bank-Formater the compound library was prepared and then screened with the three different available filters A, B and C. All the analysed compounds resulted “accepted” by the software.

### Chromatographic separation and HPLC

A sample of the mixture (DMSO) was diluted in CH_3_CN (2.0 mg/mL) and injected (20 µL) after filtration. Chromatographic run was performed on Equivalence 3 C18 column (ACE EQV-8977: 150 × 4.6 mm; 5 µm) using linear gradient elution for 23 min with a mobile phase of 0.1% v/v formic acid in H_2_O and 0.1% v/v formic acid in CH_3_CN (from 0/100 to 50/50 v/v in 23 min) at the flow rate of 0.6 mL/min. Analytes B and C were eluted after compound **1** (eluate A, t_R_ = 12.40 min), with retention times of 13.34 and 13.74 min respectively. Eluates from 13.10 to 13.90 min were collected and tested. Identification of the two main components of the fraction was performed through in-depth MS analysis. With the same method, the retention time of each pure synthesized compound (**2**–**5**) was determined and it is reported in Table 4.

**Table 4:** Mean retention times of the synthesized compounds.

Compound	Mean t_R_ ± SD (min)
**2**	13.41 ± 0.032
**3**	14.39 ± 0.028
**4**	13.84 ± 0.031
**5**	14.30 ± 0.029

### Preliminary characterization studies

Chromatography-mass spectrometry (LC-MS) system consisted of an Agilent 1100 series liquid chromatograph system (Agilent Technologies, Palo Alto, CA) including a vacuum solvent degassing unit, a binary high-pressure gradient pump, an UV detector, and an 1100 MSD model VL benchtop mass spectrometer with API-ES interface. The UV detector was set at 210 nm. The Agilent 1100 series MSD single-quadrupole instrument was equipped with the orthogonal spray API (Agilent Technologies, Palo Alto, CA). Nitrogen was used as nebulizer gas and drying gas (350 °C). The LC-API-MS determination was performed by operating the MSD in the positive ion mode. Mass spectra were acquired over the scan range 105–1500 *m*/*z* using a step size of 0.1 u. The nebulizer gas, the drying gas, the capillary voltage, and the vaporizer temperature were set at 40 psi, 9 L/min, 3000 V and 350 °C, respectively. For the fragmentation study the fragmentor voltage was set in the range 30–200 mV.

### Accurate mass and fragmentation studies

The accurate masses were measured by the LTQ-Orbitrap XL (Thermo Scientific, Bremen, Germany) mass spectrometer interfaced with an electrospray ionization (ESI) source characterized by a spray voltage of 4.5 kV and nitrogen as sheat gas (10 a.u.). The resolution of accurate masses is 30000. MS/MS spectra were recorded with an isolation window of 2 mass units, collision energy of 15, 16 or 17 V and helium as collision gas.

All data were processed using Xcalibur (Thermo Scientific, Bremen, Germany). The elementar composition tool was used to calculate the proposed chemical formulas, (ring and double bond) RDB values and the difference between the theoretical and experimental *m/z* for product ions.

#### Synthetic procedures and characterizations of compounds **1–5**

For the synthetic procedures and characterizations of compounds **6–25** see Supplementary Information.

#### *N*‐{8‐[(8‐carbamimidamidooctyl)amino]octyl}‐*N*′‐(cyclopropylmethyl)guanidine (**1**)

Compound **10** (10.0 mg, 0.12 mmol) was dissolved in dry DCM (1.8 mL) and TFA (10%, 0.2 mL) was added. The reaction mixture was stirred at room temperature for 7.5 h. Then the solvent was evaporated and the crude product was dissolved and evaporated several times first with CH_3_OH to remove TFA residue and then with Et_2_O to precipitate the desired compound. No further purification followed; the product was obtained as a colourless oil. ^**1**^ **H NMR** (CD_3_OD, 400 MHz): δ 0.26 (d, *J* = 4.8 Hz, 2 H); 0.58 (d, *J* = 7.2 Hz, 2 H); 1.05 (s, 1 H); 1.28 (s, 24 H); 2.96–3.00 (m, 2 H); 3.05 (d, *J* = 7.2 Hz, 2 H); 3.12–3.20 (m, 4 H). ^**13**^ **C NMR** (CD_3_OD, 100 MHz): δ 2.4; 9.5; 25.8; 26.0; 26.1; 28.3; 28.4; 28.6; 29.2; 29.4; 40.9; 41.1; 45.8; 46.9; 48.1; 157.0; 159.7. **LC-MS** *m/z* (ES+) = 410.1 [M + H]^+^; 205.5 [M + 2 H]^2+^ **YIELD:** quantitative.

#### 1,3‐bis(8‐carbamimidamidooctyl)‐1,3‐bis({8‐[*N*′‐(cyclopropylmethyl)carbamimidamido]octyl})urea (**2**)

The same procedure for the synthesis of compound **1** was followed. ^**1**^ **H NMR** (CD_3_OD, 400 MHz): δ 0.23–0.30 (m, 4 H); 0.53–0.61 (m, 4 H); 1.01–1.10 (m, 2 H); 1.31–1.45 (m, 32 H); 1.48–1.55 (m, 8 H); 1.55–1.63 (m, 8 H); 3.05 (d, *J* = 6.8 Hz, 4 H); 3.08–3.21 (m, 12 H); 3.28–3.32 (m, 4 H). ^**13**^ **C NMR** (CD_3_OD, 100 MHz): δ 2.4; 9.5; 26.2; 26.5; 27.5; 28.4; 28.4; 28.8; 28.9; 29.2; 41.0; 41.1; 45.8; 46.9; 47.1; 47.3; 47.5; 47.7; 47.9; 48.1; 155.6; 165.4. **LC-MS** *m/z* (ES+) = 845.8 [M + H]^+^; 423.1 [M + 2 H]^2+^; 282.5 [M + 3 H]^3+^; 212.1 [M + 4 H]^4+^ **YIELD:** quantitative.

#### 1-(8-carbamimidamidooctyl)-1-[8-[[*N*-(cyclopropylmethyl)carbamimidoyl]amino]octyl]-3-[*N*-[8-[8-[[*N*-(cyclopropylmethyl)carbamimidoyl]amino]octylamino]octyl]carbamimidoyl]urea (**3**)

The same procedure for the synthesis of compound **1** was followed. ^**1**^ **H NMR** (CD_3_OD, 400 MHz): δ 0.23–0.27 (m, 4 H); 0.54–0.58 (m, 4 H); 1.00-1-10 (m, 2 H); 1.26–1.40 (m, 32 H); 1.44–1.62 (m, 16 H); 2.52 (t, *J* = 8.0 Hz, 4 H); 3.03 (d, *J* = 8.0 Hz, 4 H); 3.11–3.20 (m, 12 H). ^**13**^ **C NMR** (CD_3_OD, 100 MHz): δ 3.4; 11.2; 26.7; 27.1; 28.8; 29.3; 30.4; 30.6; 41.5; 42.0; 42.3; 44.7; 49.9; 155.4; 155.8; 157.8; 158.0. **LC-MS** *m/z* (ES+) = 845.8 [M + H]^+^; 423.1 [M + 2 H]^2+^; 282.5 [M + 3 H]^3+^; 212.1 [M + 4 H]^4+^ **YIELD**: quantitative.

#### 3‐(8‐carbamimidamidooctyl)‐1‐{8‐[({[(8‐carbamimidamidooctyl)({8‐[*N*′‐(cyclopropylmethyl)carbamimidamido]octyl})carbamoyl]amino}methanimidoyl)amino]octyl}‐1,3‐bis({8‐[*N*′‐(cyclopropylmethyl)carbamimidamido]octyl})urea (**4**)

The same procedure for the synthesis of compound **1** was followed. ^**1**^ **H NMR** (CD_3_OD, 400 MHz): δ 0.24–0.28 (m, 6 H); 0.56–0.59 (m, 6 H); 1.02–1.11 (m, 3 H); 1.22–1.46 (m, 48 H); 1.49–1.70 (m, 24 H); 3.00 (d, *J* = 7.2 Hz, 6 H); 3.12–3.27 (m, 24 H). ^**13**^ **C NMR** (CD_3_OD, 100 MHz): δ 3.4; 11.1; 26.6; 26.7; 28.8; 29.3; 30.4; 41.5; 41.9; 42.3; 44.7; 49.8; 50.5; 155.4; 155.8; 157.9; 158.0; 164.5. **LC-MS** *m/z* (ES+) = 641.1 [M + 2 H]^2+^; 427.5 [M + 3 H]^3+^; 320.9 [M + 4 H]^4+^ **YIELD:** quantitative.

#### 1-(8-carbamimidamidooctyl)-1-[8-[[*N*-(cyclopropylmethyl)carbamimidoyl]amino]octyl]-3-[*N*-[8-[8-[[*N*-(cyclopropylmethyl)carbamimidoyl]amino]octyl-[[*N*-[8-[8-[[*N*-(cyclopropylmethyl)carbamimidoyl]amino]octylamino]octyl]carbamimidoyl]carbamoyl]amino]octyl]carbamimidoyl]urea (**5**)

The same procedure for the synthesis of compound **1** was followed. ^**1**^ **H NMR** (CD_3_OD, 400 MHz): δ 0.23–0.27 (m, 6 H); 0.54–0.59 (m, 6 H); 1.08–1.15 (m, 3 H); 1.26–1.37 (m, 48 H); 1.50–1.70 (m, 24 H); 2.95 (t, *J* = 8.0 Hz, 4 H) 3.03 (d, *J* = 4.0 Hz, 6 H); 3.13–3.33 (m, 20 H). ^**13**^ **C NMR** (CD_3_OD, 100 MHz): δ 3.5; 11.2; 26.7; 27.1; 28.8; 29.3; 30.4; 30.6; 41.6; 41.9; 42.3; 44.7; 50.0; 155.5; 157.8; 158.0. **LC-MS** *m/z* (ES+) = 641.1 [M + 2 H]^2+^; 427.5 [M + 3 H]^3+^; 320.9 [M + 4 H]^4+^ **YIELD:** quantitative.

#### Antibacterial susceptibility testing

Bacterial strains, including representatives of both Gram-positive and Gram-negative bacteria, were obtained from the ATCC or CCUG culture collections. Compounds were re-suspended in DMSO at a final concentration of 50 or 100 mg/mL and subsequently diluted in the culture medium. The minimum inhibitory concentration (MIC) and the minimum bactericidal concentration (MBC) of the compounds were determined using the micro-dilution broth method using Mueller-Hinton broth as recommended by the Clinical Laboratory Standards Institute (CLSI)^48^. Bacterial inoculum was 5 × 10^4^ CFU/well. MICs were recorded after 18 hours of incubation at 35 °C after visual observation of solution transparency. MIC and MBC values are reported as median values and were obtained from experiments performed at least in triplicate.

The haemolytic activity of selected compounds was estimated using the method described by Bechlars *et al* ^49^. 10 µL of compound (concentrations, 0.25 to 25 mg/mL) were spotted on the surface of a blood agar plate. Triton X-100 was used as a positive haemolysis control.

## Electronic supplementary material

SUPPLEMENTARY INFORMATION
