# Supplementary material for: Identification, synthesis and biological activity of alkyl-guanidine oligomers as potent antibacterial agents
Source: Sci Rep. 2017 Aug 15;7:8251. doi: 10.1038/s41598-017-08749-6 (PMC5557985; doi:10.1038/s41598-017-08749-6)
Supplement: Supplementary file 1 — SUPPLEMENTARY INFORMATION [file 41598_2017_8749_MOESM1_ESM.pdf]

SUPPLEMENTARY INFORMATION FOR:

Identification, synthesis and biological activity of alkyl-guanidine oligomers as potent antibacterial agents

*Zamperini, C.[1,2], Maccari, G.[1], Deodato, D.[1], Pasero, C.[1], D'Agostino, I.[1], Orofino, F.[1], De Luca, F.[3], Dreassi, E.[1], Docquier, J.D.[2,3] and Botta, M.[1,2,4]**

[1]Department of Biotechnology, Chemistry and Pharmacy, University of Siena, I-53100 Siena, Italy

[2]Lead Discovery Siena s.r.l., Via Vittorio Alfieri 31, I-53019 Castelnuovo Berardenga, Italy

[3]Department of Medical Biotechnology, University of Siena, I-53100 Siena, Italy

[4]Sbarro Institute for Cancer Research and Molecular Medicine, Temple University, BioLife Science Building, Suite 333, 1900 North 12th Street, Philadelphia, Pennsylvania 19122, United States of America

* botta.maurizio@gmail.com

TABLE OF CONTENTS

MS spectra integrated from the original batch HPLC

- **Supplementary Figure S1.** MS spectra integrated from the original batch HPLC using low fragmentor energy (30 mV).

Extracted-ions chromatogram of the original batch (XIC).

- **Supplementary Figure S2.** Extracted-ions chromatogram of the original batch (XIC) obtained from a chromatographic run conducted at 30 mV.
- **Supplementary Figure S3.** Extracted-ions chromatogram of the original batch (XIC) obtained from a chromatographic run conducted at 100 mV.

MS^n experiment spectra.

- **Supplementary Figure S4.** Zoom of MS^2 spectrum of dimer (845.7 *m/z*).
- **Supplementary Figure S5.** Zoom of MS^3 spectrum of dimer (845.7 → 803.9 *m/z*).
- **Supplementary Figure S6.** Zoom of MS^4 spectrum of dimer (845.7 → 803.9 → 707.8 *m/z*).
- **Supplementary Figure S7.** MS^2 spectrum of trimer (1281.1 *m/z*).

Synthetic procedures and characterizations for compounds 6-25.

Proposed mechanism of urea moiety formation in compounds 2 and 4.

- **Supplementary Figure S8.** First putative mechanism of urea moiety formation involving atmospheric carbon dioxide.
- **Supplementary Figure S9.** Second putative mechanism of urea moiety formation involving a macrocyclic byproduct.

Kill curve assay of compound 2.

- **Supplementary Figure S10.** Kill curve of compound **2** on *S. aureus* ATCC 25923.

Supplementary Table S1. Molecular formula strings of compounds **1-25**

Supplementary References

MS spectra integrated from the original batch HPLC. The MS spectra A, B and C (**Supplementary Figure S1**) reporting *m/z* values of monomer (**1**), dimer and trimer respectively were obtained by the integration process of the MS signals corresponding to the three main UV peaks in the chromatogram of the original batch. In each spectrum pseudomolecolar and/or multiple charged ions were highlighted.

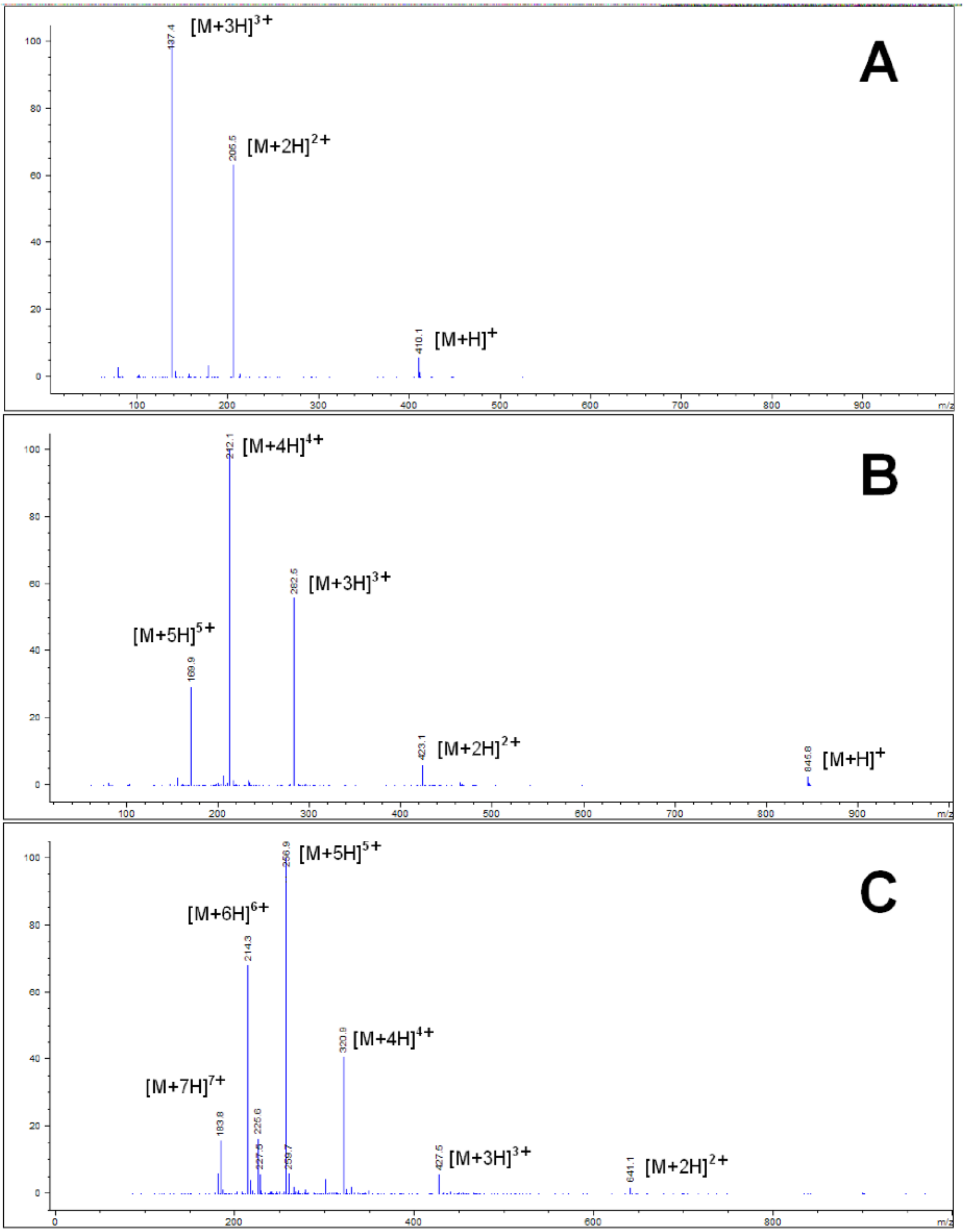

Supplementary Figure S1. MS spectra integrated from the original batch HPLC using low fragmentor energy (30 mV).

Extracted-ions chromatogram of the original batch (XIC). The extracted-ion chromatograms (XIC) reported in **Supplementary Figure S2** and **S3** showed *m/z* values representing monomeric (**1**) (A), dimeric (B) and trimeric (C) analytes recovered from the entire data set for the chromatographic run of a sample of the original batch. The chromatographic profiles were obtained using low (30 mV) and medium (100 mV) fragmentor voltage in **Supplementary Figure S2** and **S3** respectively. Total ion current (TIC) chromatogram was shown in the first line of each figure. At low fragmentation energy *m/z* values of monomer (**1**), dimer and trimer as pseudomolecolar or multiple charged ions univocally corresponded to the first, second and third TIC signals respectively (**Supplementary Figure S2**). While at higher fragmentation energy the *m/z* values characteristic of monomer (**1**) (410.1 and 205.5 *m/z*) were detected also in correspondence of the other TIC signals (B and C) (**Supplementary Figure S3**), thus demonstrating that the other analytes derived from the monomer (**1**).

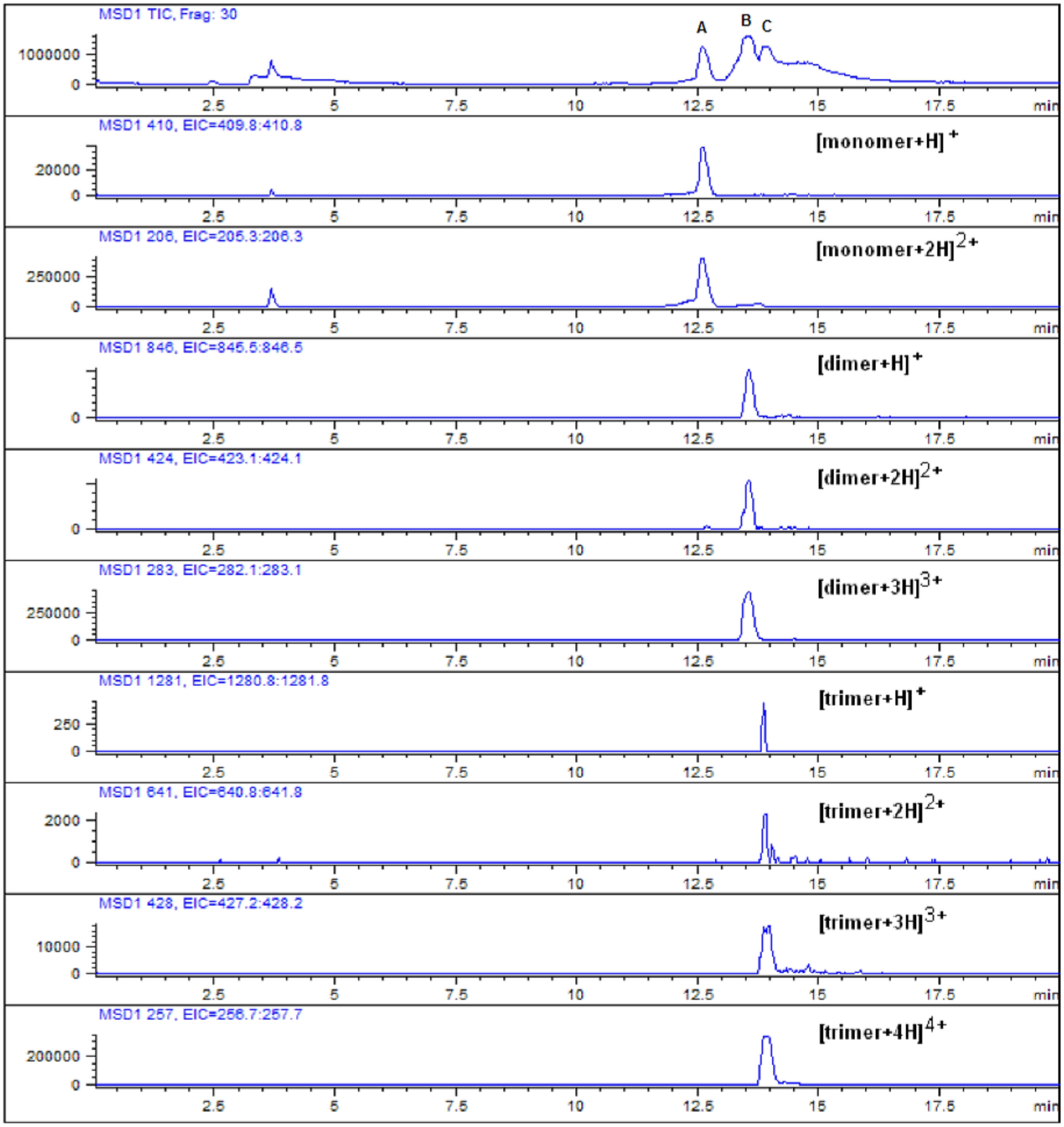

Supplementary Figure S2. Extracted-ions chromatogram of the original batch (XIC) obtained from a chromatographic run conducted at 30 mV.

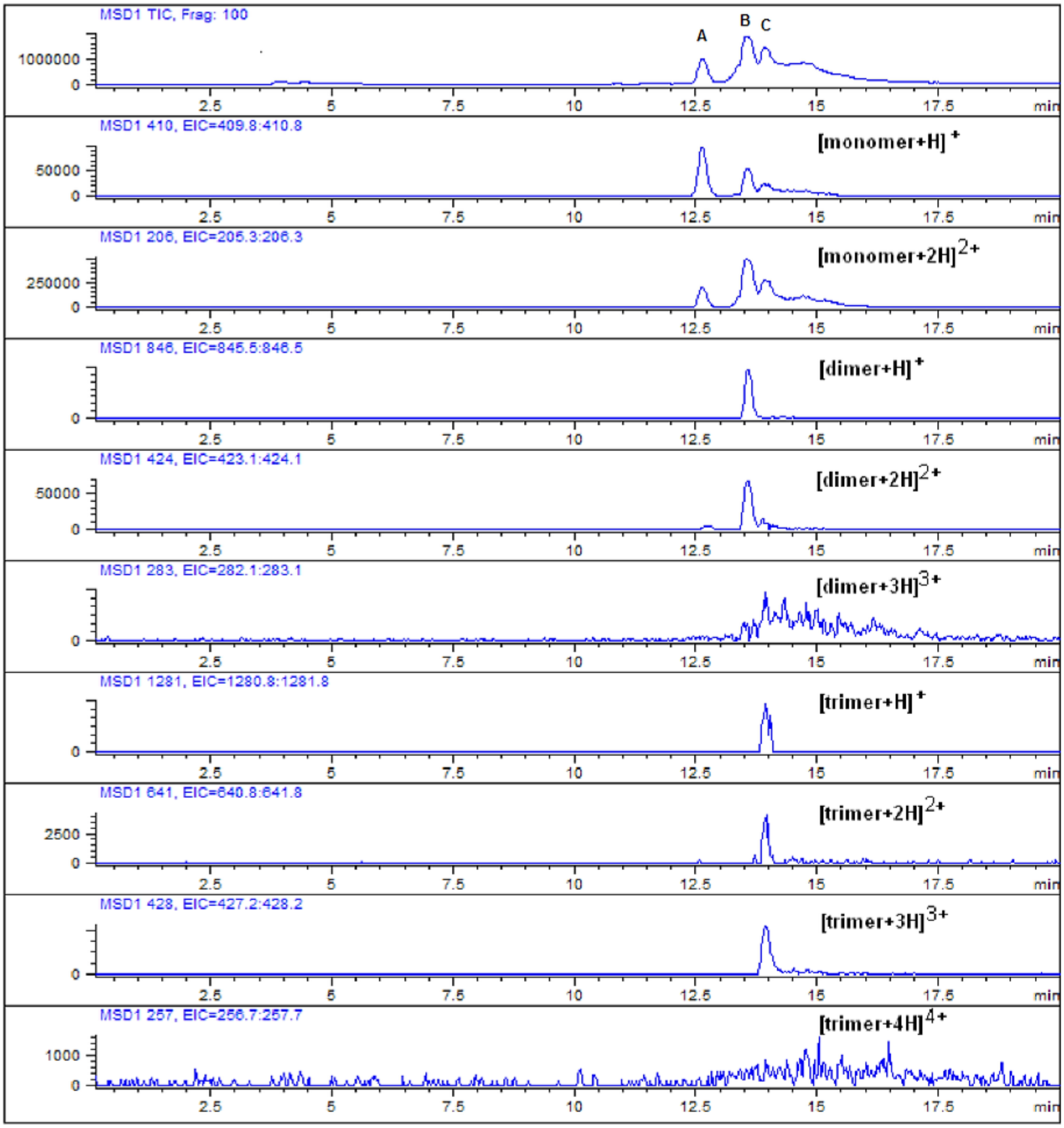

Supplementary Figure S3. Extracted-ions chromatogram of the original batch (XIC) obtained from a chromatographic run conducted at 100 mV.

MS^n experiment spectra. The MS^2 spectrum obtained from the precursor ion 845.7 *m/z* (dimer) showed the formation of several product ions, in particular 803.9 *m/z* corresponding to the loss of a methanediimmine fragment (42.0 *m/z*) (**Supplementary Figure S4**).

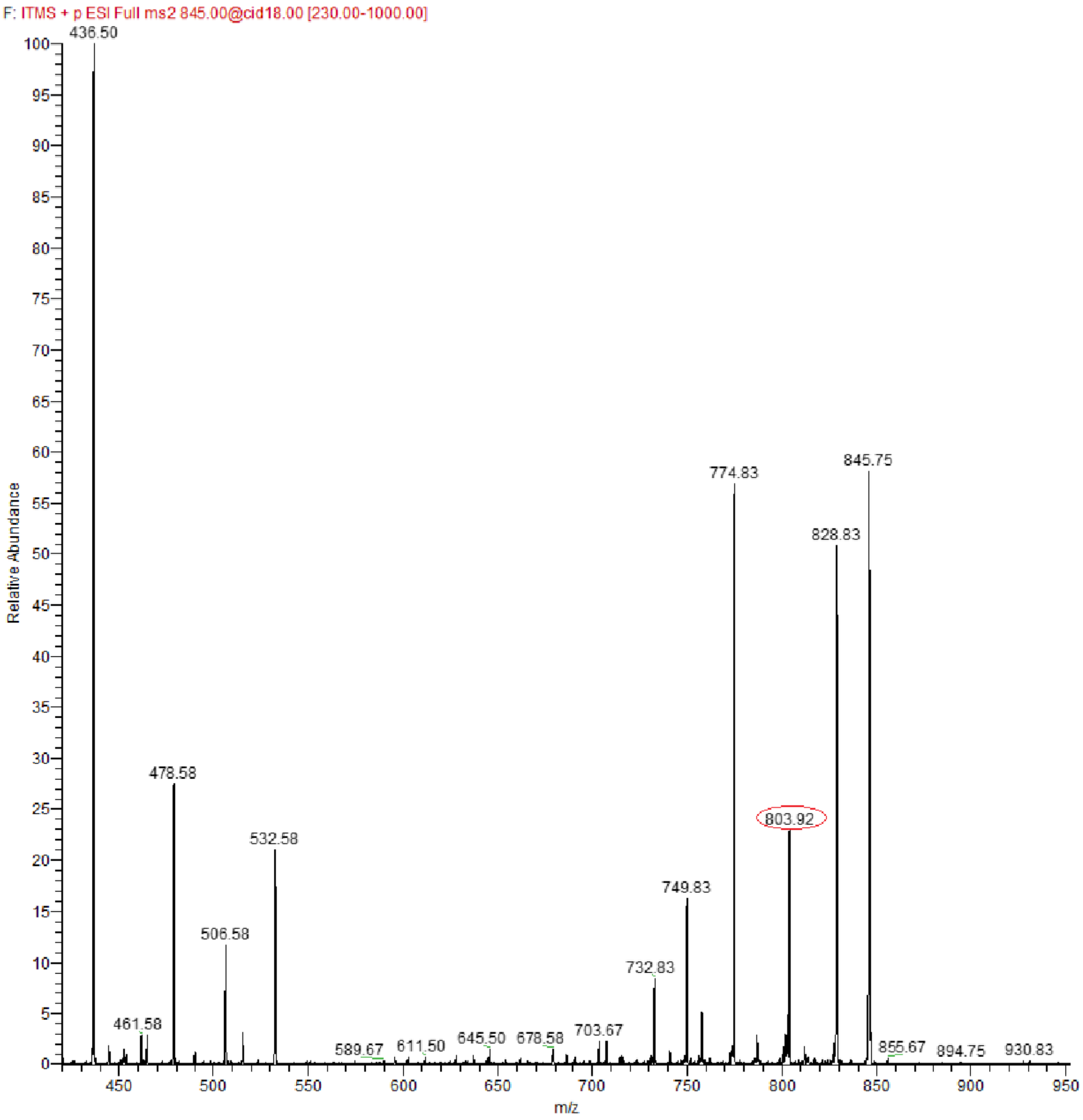

Supplementary Figure S4. Zoom of MS^2 spectrum of dimer (845.7 *m/z*).

From the entrapment of 803.9 *m/z* obtained from 845.7 *m/z* (dimer) and its following fragmentation, MS^3 spectrum showed 707.8 *m/z* as one of the main signals, which derived from the loss of *N*-(cyclopropylmethyl)-cyanamide fragment (96.1 *m/z*) (**Supplementary Figure S5**).

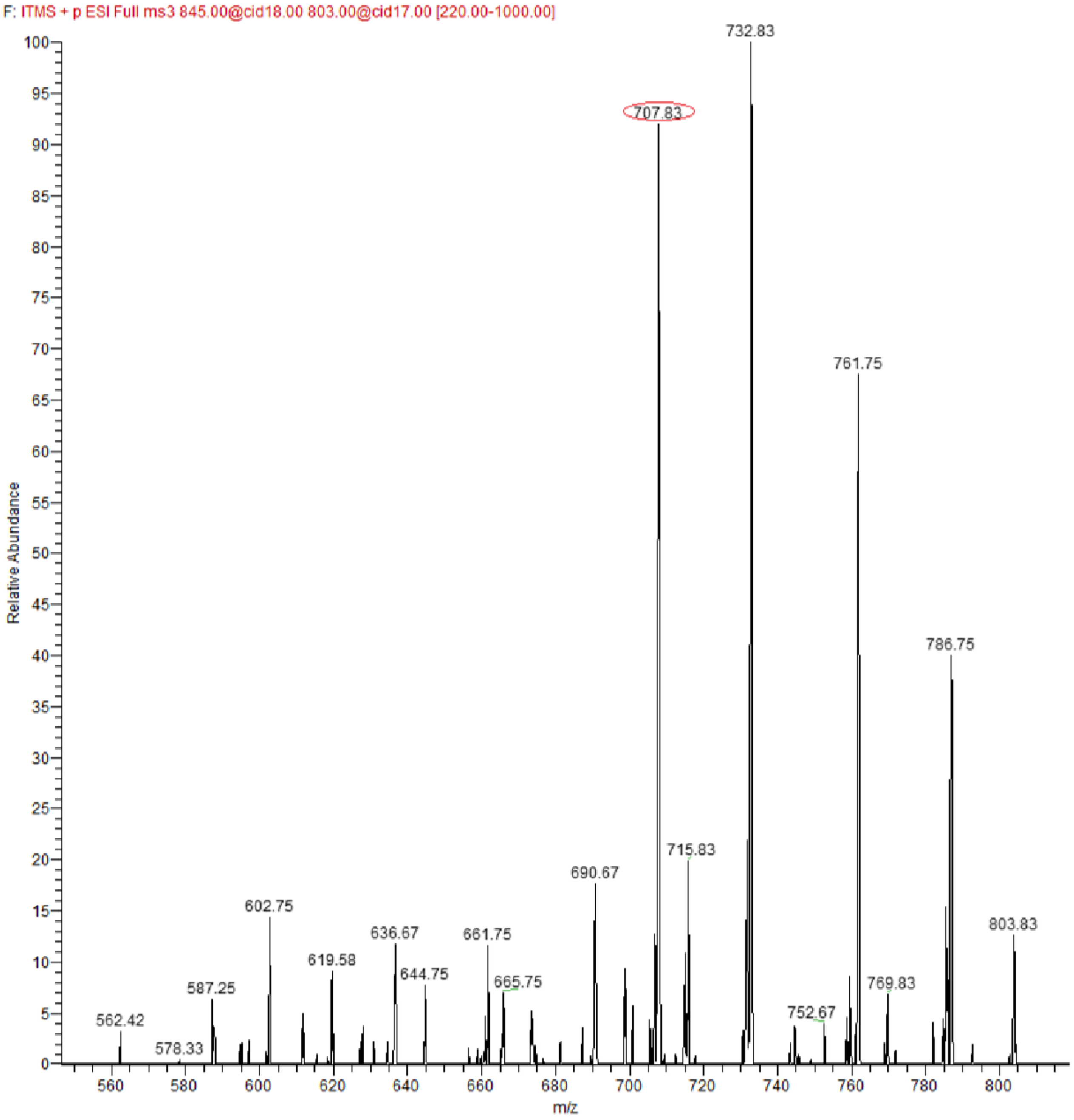

Supplementary Figure S5. Zoom of MS^3 spectrum of dimer (845.7 → 803.9 *m/z*).

In MS[4] spectrum of dimer (845.7 → 803.9 → 707.8 *m/z*), the signal at 665.8 *m/z* was detected. It was due to the loss of another methanediimmine fragment (42.0 *m/z*), characteristic of only the symmetric isomers (**Supplementary Figure S6**).

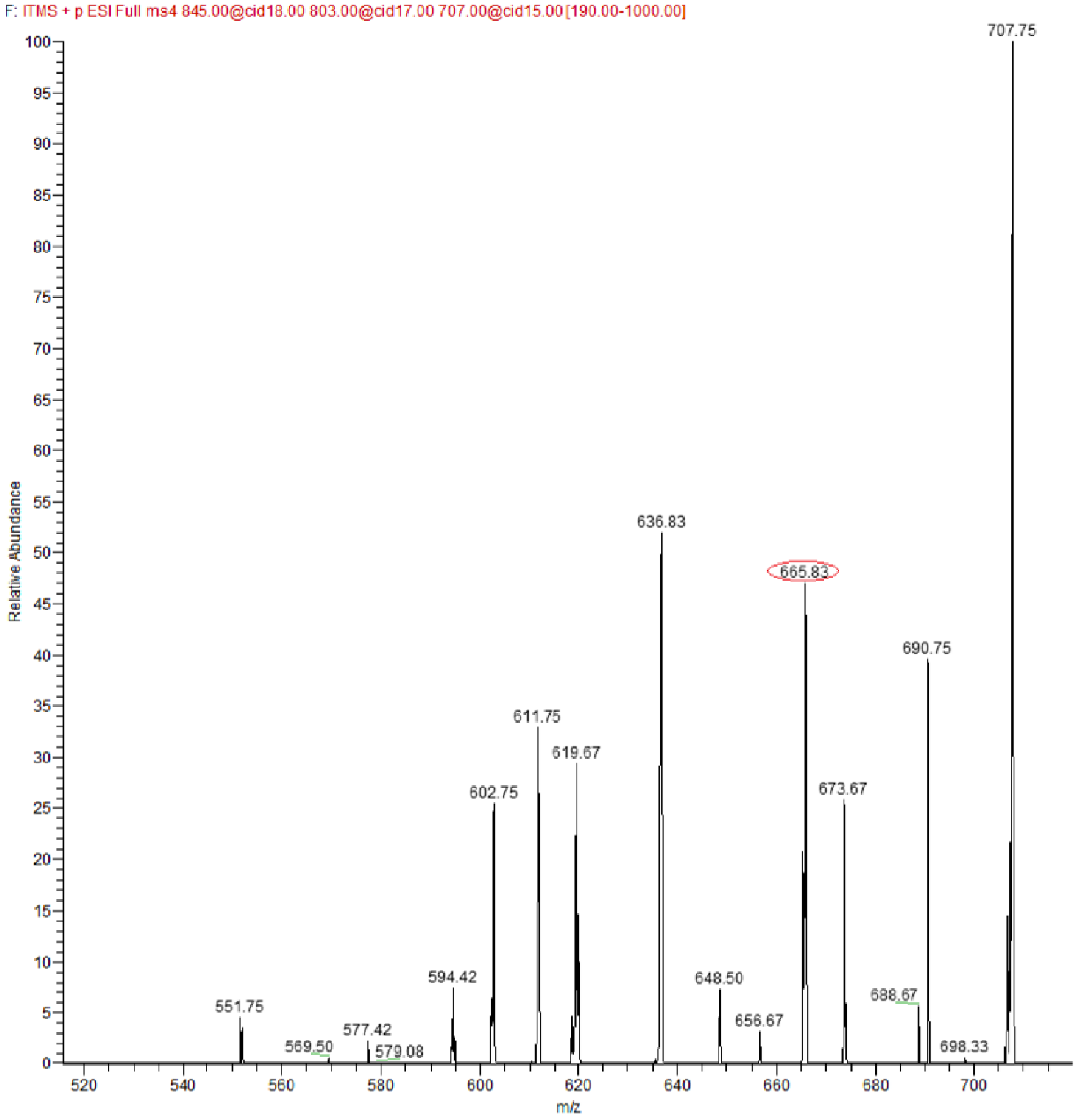

Supplementary Figure S6. Zoom of MS[4] spectrum of dimer (845.7 → 803.9 → 707.8 *m/z*).

From the isolation through the ion trap of the precursor ion 1281.1 *m/z* (trimer), MS^2 spectrum showed 845.8 *m/z* and 410.5 *m/z* as the main signals, corresponding respectively to dimer and monomer (**Supplementary Figure S7**).

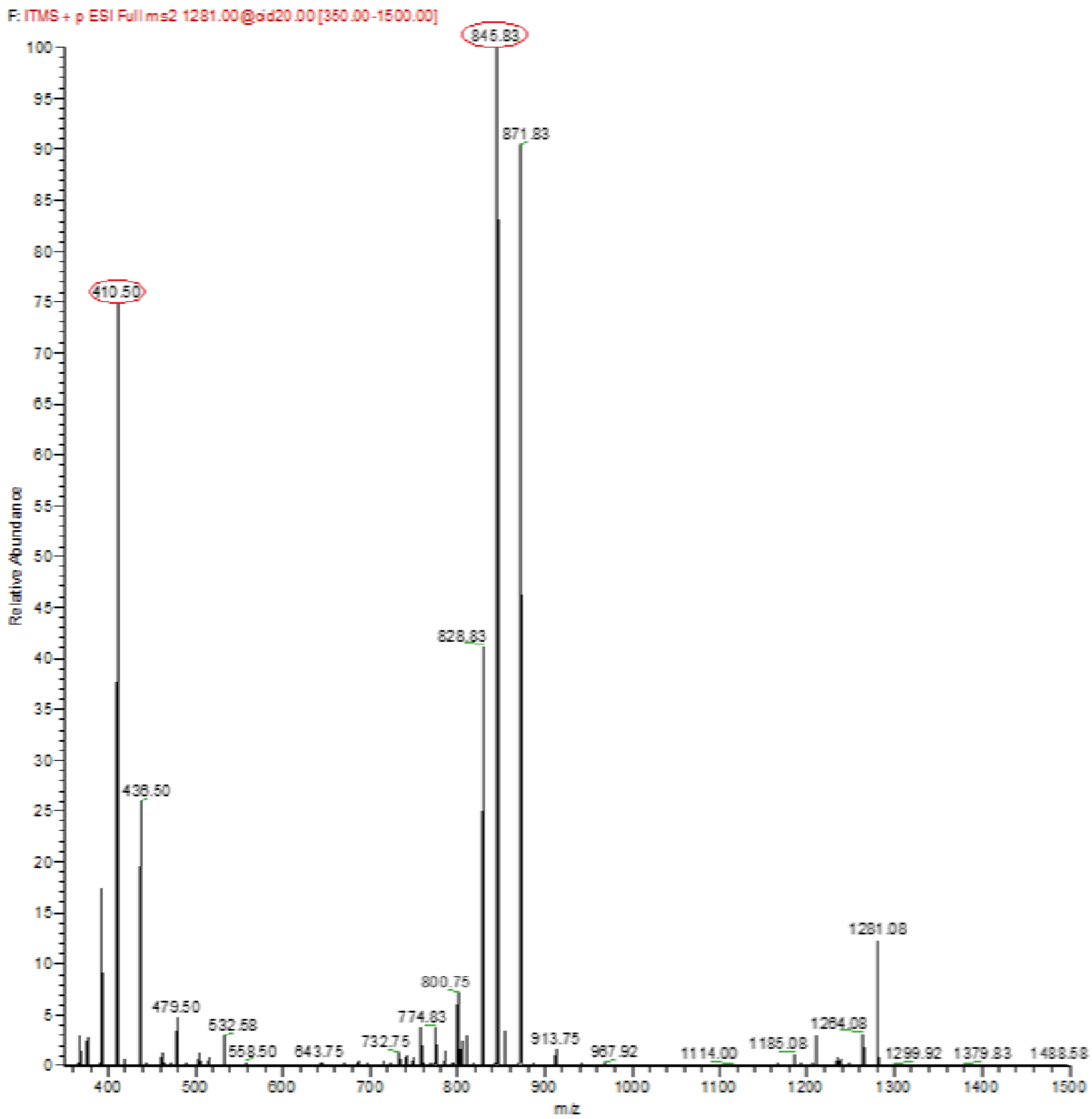

Supplementary Figure S7. MS^2 spectrum of trimer (1281.1 *m/z*).

Synthetic procedures and characterizations for compounds 6-25.

1-azido-8-bromoctane (6) To a solution of 1,8-dibromooctane (6.0 mL, 32.60 mmol) in DMF (60.0 mL), sodium azide (1.21 g, 19.56 mmol) was added and the reaction mixture was stirred at 50 °C overnight. After cooling, the reaction mixture was concentrated under reduced pressure and treated with H_2O (20.0 mL). The aqueous phase was extracted three times with AcOEt (20.0 mL), the combined organic layers were washed with Brine (60.0 mL), dried over Na_2SO_4 and evaporated under reduced pressure. The crude product was purified by flash column chromatography (silica gel) (Hexane), affording the product as a yellowish oil. **^{1}H NMR** ($CDCl_3$, 400 MHz): δ 1.26-1.38 (m, 6H); 1.40-1.46 (m, 2H); 1.54-1.64 (m, 2H); 1.80-1.89 (m, 2H); 3.21-3.28 (m, 2H); 3.37-3.42 (m, 2H). **^{13}C NMR** ($CDCl_3$, 100 MHz): δ 26.5; 27.9; 28.5; 28.7; 28.8; 32.6; 33.7; 51.3. **YIELD:** 72%

***N'*-(8-aminooctyl)-*N,N"*-di-Boc-guanidine (7)** To a solution of 1,8-diaminooctane (6.00 g, 41.67 mmol) in CH_3CN/CH_3OH 9/1 (75.0 mL), 1,3-Bis(tert-butoxycarbonyl)-2-methyl-2-thiopseudourea (4.04 g, 13.89 mmol) in CH_3CN/CH_3OH 9/1 (75.0 mL) was added and the reaction mixture was stirred at 40 °C. DIPEA (3.0 mL, 17.09 mmol) was added to the reaction mixture and it was stirred at 40 °C overnight. Then the reaction mixture was concentrated under reduced pressure and the crude product was purified by flash column chromatography (silica gel) ($CH_3CN/CH_3OH/Et_3N$ 8/2/1), affording the product as a pale-yellow oil. **^{1}H NMR** ($CDCl_3$, 400 MHz): δ 1.23-1.38 (m, 12H); 1.49 (s, 18H); 2.67 (t, *J* = 7.0 Hz, 2H); 3.35-3.45 (m, 2H); 8.28 (br, 1H); 11.49 (br, 1H). **^{13}C NMR** ($CDCl_3$, 100 MHz): δ 26.6; 26.7; 26.9; 27.3; 27.5; 27.8; 28.0; 28.3; 28.6; 29.3; 30.1; 30.4; 40.8; 83.2; 153.4; 156.3; 163.7. **LC-MS** *m/z* (ES+) = 387.0 $[M + H]^+$ **YIELD:** 89%

***N'*-{8-[(8-azidooctyl)amino]octyl}-*N*,N''-di-Boc-guanidine (8)** To cesium hydroxide monohydrate (266.0 mg, 1.59 mmol) and molecular sieves (600.0 mg) dry DMF (5.0 mL) was added under nitrogen atmosphere. The mixture was stirred for 10 min. A solution of **7** (612.0 mg, 1.59 mmol) in dry DMF (5.0 mL) was added and the mixture was stirred for further 30 min. Then **6** (297.0 mg, 1.27 mmol) was added and the reaction mixture was stirred at room temperature overnight. The mixture was diluted with AcOEt (10.0 mL), filtered from the solid, washed and concentrated under vacuum. The residue was treated with NaOH 1N (20.0 mL) and extracted three times with AcOEt (20.0 mL). The combined organic phases were washed with H_2O (60.0 mL), LiCl 5% (60.0 mL) and Brine (60.0 mL). The crude product was purified by flash column chromatography (silica gel) (DCM/CH_3OH 9/1), affording the product as a yellowish oil. **^{1}H NMR** ($CDCl_3$, 400 MHz): δ 1.25-1.35 (m, 24H); 1.44 (s, 18H); 2.52 (t, *J* = 7.0 Hz, 4H); 3.19 (t, *J* = 6.8 Hz, 2H); 3.34 (q, 2H, *J* = 5.6 Hz); 8.23 (br, 1H); 11.50 (br, 1H). **^{13}C NMR** ($CDCl_3$, 100 MHz): δ 26.7; 27.0; 28.4; 29.3; 30.1; 30.3; 30.5; 41.9; 49.9; 50.0; 79.9; 84.6; 153.7; 158.0; 160.4. **LC-MS** *m/z* (ES+) = 540.1 $[M + H]^+$ **YIELD:** 43%

***N'*-{8-[(8-aminooctyl)amino]octyl}-*N,N''*-di-Boc-guanidine (9)** Compound **8** (334.1 mg, 0.62 mmol) was dissolved in *i*-PrOH (40.0 mL) and Pd/C 10% (127.8 mg, 0.12 mmol) was added. The reaction mixture was subjected to 3 cycles of vacuum followed by flash of H_2, and it was stirred under H_2 atmosphere for 4 h. The reaction mixture was diluted with CH_3OH (100.0 mL) and filtered through a plug of celite. The filtrate was evaporated under reduced pressure and the compound was obtained without any further purification as a yellowish oil. **^{1}H NMR** (CD_3OD, 400 MHz): δ 1.30-1.40 (m, 24H); 1.52 (s, 18H); 2.62 (t, *J* = 7.6 Hz, 4H); 3.28-3.32 (m, 2H); 3.32-3.38 (m, 2H). **^{13}C NMR** (CD_3OD, 100 MHz): δ 26.3; 26.4; 26.8; 27.1; 28.6; 28.7; 29.0; 29.0; 31.6; 40.3; 40.8; 49.1; 78.8; 83.0; 152.8; 156.1; 163.1. **LC-MS** *m/z* (ES+) = 514.0 $[M + H]^+$ **YIELD:** quantitative

***N'*-(cyclopropylmethyl)-*N*-(8-{[8-(*N',N''*-di-Boc-carbamimidamido)octyl]amino}octyl)-*N',N''*-di-Boc-guanidine (10)** A solution of *N,N'*-Di-Boc-*N*- methylcyclopropyl-pyrazole-1-carboxamidine (262.1 mg, 0.72 mmol) in THF (5.8 mL) was added to **9** (310.0 mg, 0.60 mmol). DIPEA (0.1 mL, 0.60 mmol) was added and the reaction mixture was stirred at room temperature

overnight. Then the mixture was diluted with $NaHCO_3$ s.s. (10.0 mL), the aqueous phase was extracted three times with AcOEt (15.0 mL). The combined organic layers were washed with Brine (45.0 mL), dried over Na_2SO_4 and concentrated under reduced pressure. The crude product was purified by flash column chromatography (silica gel) (DCM/CH_3OH 9/1), affording the product as a pale-yellow oil. **^{1}H NMR** ($CDCl_3$, 400 MHz): δ 0.24 (d, *J* = 4.8 Hz, 2H); 0.45 (d, *J* = 7.6 Hz, 2H); 1.00-1.10 (m, 1H); 1.25-1.41 (m, 16H); 1.49 (s, 36H); 1.50-1.60 (m, 8H); 2.58 (t; *J* = 7.2 Hz, 4H); 3.25-3.35 (m, 2H); 3.39 (q, *J* = 6.5 Hz, 2H); 3.51-3.58 (m, 2H); 8.28 (br, 1H). **^{13}C NMR** ($CDCl_3$, 100 MHz): δ 3.4; 10.4; 26.1; 26.6; 26.7; 27.9; 28.1; 28.2; 28.8; 29.0; 29.0; 40.7; 44.0; 47.6; 52.0; 79.0; 81.8; 82.9; 153.2; 156.0; 163.5. **LC-MS** *m/z* (ES+) = 809.9 $[M + H]^+$; 405.5 $[M + 2H]^{2+}$. **YIELD:** 70%

***N*-{8-[*N'*-(cyclopropylmethyl)-*N'*,*N''*-di-Boc-carbamimidamido]octyl}-*N*-[8-(*N'*,*N''*-di-Boc-carbamimidamido)octyl]carbamoyl chloride (11)** To a solution of compound **10** (97.0 mg, 0.12 mmol) in dry THF (5.0 mL) DIPEA (0.021 mL, 0.12 mmol) and triphosgene (29.7 mg, 0.10 mmol) were added at 0 °C under nitrogen atmosphere. The mixture was stirred 10 min, then ice bath was removed and the mixture was left at room temperature for 1 h. Then $NaHCO_3$ s.s. (5.0 mL) was added to the reaction mixture and it was stirred for 10 min. The aqueous phase was extracted three times with AcOEt (5.0 mL); the combined organic layers were washed with Brine (15.0 mL), dried over Na_2SO_4 and concentrated. The solvent was removed under reduced pressure and the crude product was purified by flash column chromatography (silica gel) (DCM/CH_3OH 98/2), affording the product as a colourless oil. **^{1}H NMR** ($CDCl_3$, 400 MHz): δ 0.24 (d, *J* = 4.8 Hz, 2H); 0.44 (d, *J* = 8.0 Hz, 2H); 0.81-0.91 (m, 2H); 1.25-1.40 (m, 16H); 1.49 (s, 36H); 1.53-1.68 (m, 8H); 3.28-3.32 (m, 4H); 3.33-3.41 (m, 4H); 3.49-3.60 (m, 2H); 8.27 (br, 1H); 11.49 (br, 1H). **^{13}C NMR** ($CDCl_3$, 100 MHz): δ 3.5; 10.5; 14.0; 22.6; 24.7; 26.5; 26.6; 26.8; 27.4; 28.0; 28.1; 28.2; 28.3; 28.8; 29.0; 29.6; 31.8; 33.7; 40.8; 43.8; 49.8; 51.1; 52.1; 79.1; 81.9; 82.9; 85.2; 131.6; 148.9; 153.2; 156.0; 163.5. **LC-MS** *m/z* (ES+) = 872.2 $[M + H]^+$; 436.5 $[M + 2H]^{2+}$ **YIELD:** 72%

1,3-bis({8-[N'-(cyclopropylmethyl)-*N'*,*N''*-di-Boc-carbamimidamido]octyl})-3-[8-(*N'*,*N''*-di-Boc-carbamimidamido)octyl]-1-[8-(*N''*-Boc-carbamimidamido)octyl]urea (12) To a solution of **11** (69.7mg, 0.08 mmol) in dry DCM (3.4 mL) a solution of **10** (97.0 mg, 0.12 mmol) in dry DCM (3.4 mL) was added. Then DIPEA (0.01 mL, 0.08 mmol) and sodium iodide (catalytic amount) were added and the reaction mixture was stirred at 40° C for 48 h. After cooling, AcOEt (5.0 mL) and NaOH 1N (10.0 mL) were added to the reaction mixture and it was stirred for 10 min. The aqueous phase was extracted three times with AcOEt (10.0 mL) and the combined organic layers were washed with Brine (30.0 mL), dried over Na_2SO_4 and concentrated under reduced pressure. The crude product was purified by flash column chromatography (silica gel) (DCM/CH_3OH 98/2), affording the product as a yellowish oil. **^{1}H NMR** ($CDCl_3$, 400 MHz): δ 0.26 (d, *J* = 4.8 Hz, 4H); 0.43 (d, *J* = 7.6 Hz, 4H); 0.99-1.09 (m, 2H); 1.18-1.39 (m, 48H); 1.48 (s, 72H); 3.01-3.09 (m, 8H); 3.28-3.32 (m, 4H); 3.35-3.43 (m, 4H); 3.49-3.58 (m, 4H); 8.26 (br, 2H); 11.49 (br, 2H). **^{13}C NMR** ($CDCl_3$, 100 MHz): δ 3.4; 10.5; 26.7; 26.9; 27.0; 28.0; 28.1; 28.2; 28.9; 29.2; 29.5; 40.8; 43.8; 48.2; 48.3; 52.1; 79.0; 81.8; 82.9; 153.2; 156.0; 163.5; 165.2. **LC-MS** *m/z* (ES+) = 823.5 $[M + 2H]^{2+}$; 549.4 $[M + 3H]^{3+}$ **YIELD:** 60%

***N'*-(8-{[(4-methoxyphenyl)methyl]amino}octyl)-*N*,*N''*-di-Boc-guanidine (13)** To a solution of **7** (2.990 g, 7.75 mmol) in CH_3OH (49.0 mL), *p*-Anisaldehyde (1.0 mL, 8.53 mmol) was added dropwise. The reaction mixture was stirred at room temperature for 3 h. Then, $NaBH_4$ (324.1 mg, 8.53 mmol) was added at 0 °C in four portion and the mixture was stirred at 0 °C for 1 h. Then the reaction mixture was treated with NaOH 1N (50.0 mL) and the aqueous phase was extracted three times with AcOEt (50.0 mL) and the combined organic layers were washed with Brine (200.0 mL) and dried over Na_2SO_4. The solvent was removed under reduced pressure and the crude product was purified by flash column chromatography (silica gel) (DCM/CH_3OH 95/5), affording the product as a colourless oil. **^{1}H NMR** ($CDCl_3$, 400 MHz): δ 1.28 (s, 12H); 1.48 (s, 18H); 2.58 (t, *J* = 7.2 Hz, 2H); 3.38 (q, *J* = 6.5 Hz, 2H); 3.70 (s, 2H); 3.77 (s, 3H); 6.84 (d, *J* = 8.8 Hz, 2H); 7.21 (d, *J* = 8.4 Hz, 2H); 8.26 (br, 1H); 11.48 (br, 1H). **^{13}C NMR** ($CDCl_3$, 100 MHz): δ 26.7; 28,4; 29.3; 30.4; 41.9; 46.3; 49.5; 55.9; 79.8; 84.6; 114.1; 131.6; 132.5; 153.8; 158.0; 159.0; 160.4. **LC-MS** *m/z* (ES+) = 507.0 $[M + H]^+$ **YIELD:** 68%

***N′*-{8-[(8-azidooctyl)[(4-methoxyphenyl)methyl]amino]octyl}-*N,N*"-di-Boc-guanidine (14)** To a solution of **13** (380 mg, 0.76 mmol) in DMF (5.0 mL), a solution of compound **6** (193.0 mg, 0.83 mmol) in DMF (1.0 mL) was added. The reaction mixture was stirred at room temperature for 72 h. Then the reaction mixture was treated with NaOH 1N (10.0 mL) and the aqueous phase was extracted three times with AcOEt (15.0 mL) and the combined organic layers were washed with Brine (50.0 mL) and dried over Na_2SO_4. The solvent was removed under reduced pressure and the crude product was purified by flash column chromatography (silica gel) (DCM/CH_3OH 95/5), affording the product as a pale-yellow oil. **^{1}H NMR** ($CDCl_3$, 400 MHz): δ 1.22-1.28 (m, 20H); 1.47 (s, 18H); 1.48-1.60 (m, 4H); 2.35 (t; *J* = 7.4 Hz, 4H); 3.21 (t; *J* = 7.0 Hz, 2H); 3.34-3.39 (m, 2H); 3.46 (s, 2H); 3.76 (s, 3H); 6.81 (d, *J* = 8.4 Hz, 2H); 7.19 (d, *J* = 8.4 Hz, 2H); 8.26 (br, 1H); 11.48 (br, 1H). **^{13}C NMR** ($CDCl_3$, 100 MHz): δ 26.7; 27.2; 28.0; 28.2; 28.9; 29.0; 29.2; 29.2; 40.9; 51.3; 53.4; 55.1; 57.7; 79.0; 82.8; 113.4; 128.2; 129.9; 153.2; 156.0; 158.4; 163.5. **LC-MS** *m/z* (ES+) = 660.0 $[M + H]^+$ **YIELD:** 85%

***N′*-{8-[(8-azidooctyl)amino]octyl}-*N*-Boc-guanidine (15)** To a solution of **14** (300.0 mg, 0.46 mmol) in *t*-BuOH/CH_3OH 4/1 (10.1 mL), Ammonium cerium nitrate (756.2 mg, 1.38 mmol) was added and the reaction mixture was stirred at 55 °C for 5 h. After cooling, the mixture was treated with $NaHCO_3$ s.s. (10.0 mL) and the aqueous phase was extracted three times with AcOEt (10.0 mL) and the combined organic layers were washed with Brine (40.0 mL) and dried over Na_2SO_4. The solvent was removed under reduced pressure and the crude product was purified by flash column chromatography (silica gel) (DCM/CH_3OH/TEA 8/2/1), affording the product as a colourless oil. **^{1}H NMR** ($CDCl_3$, 400 MHz): δ 1.25-1.40 (m, 16H); 1.45 (s, 9H); 1.48-1.52 (m, 4H); 1.56-1.62 (m, 4H); 2.60 (t, *J* = 7.2 Hz, 4H); 3.10 (t, *J* = 7.0 Hz, 2H); 3.23 (t, *J* = 7.0 Hz, 2H); 8.28 (br, 1H); 11.49 (br, 1H). **^{13}C NMR** ($CDCl_3$, 100 MHz): δ 26.8; 27.1; 28.4; 29.3; 30.1; 30.3; 30.5; 41.8; 49.9; 50.0; 84.6; 153.7; 155.5. **LC-MS** *m/z* (ES+) = 440.3 $[M + H]^+$ **YIELD:** 33%

3-(8-azidooctyl)-1-(*N*-{8-[(8-azidooctyl)[(4-methoxyphenyl)methyl]amino]octyl}-*N′*-Boc-carbamimidoyl)-3-[8-(*N′*-Boc-carbamimidamido)octyl]urea (16) To a solution of **15** (63.0 mg, 0.10 mmol) in dry THF (1.0 mL) DIPEA (catalytic amount) was added and the reaction was stirred at reflux. Then a solution of **14** (14.0 mg, 0.03 mmol) in dry THF (0.5 mL) was added dropwise using a syringe pump during a period of 4 h. The reaction mixture was stirred at reflux for a further 2 h. After cooling, H_2O (5.0 mL) was added and the aqueous phase was extracted three times with AcOEt (5.0 mL). The combined organic layers were washed with Brine (15.0 mL), dried over Na_2SO_4 and evaporated under reduced pressure. The crude product was purified by flash column chromatography (silica gel) (DCM/CH_3OH 9/1), affording the product as a pale-yellow oil. **^{1}H NMR** ($CDCl_3$, 400 MHz): δ 1.20-1.40 (m, 32H); 1.43 (s, 9H); 1.46 (s, 9H); 1.51-1.68 (m, 16H); 2.38-2.42 (m, 2H); 2.43-2.52 (m, 2H); 3.15-3.30 (m, 10H); 3.35-3.40 (m, 2H); 3.51 (s, 1H); 3.62 (s, 1H); 3.77 (s, 3H); 6.83 (t, *J* = 8 Hz, 2H); 7.23 (t; *J* = 8 Hz, 2H); 7.96 (br, 1H). **^{13}C NMR** ($CDCl_3$, 100 MHz): δ 26.7; 27.3; 28.4; 29.3; 30.1; 30.4; 41.6; 42.0; 49.9; 55.9; 56.9; 65.0; 79.8; 84.6; 114.1; 130.9; 132.5; 149.1; 153.7; 155.4; 155.9; 158.0; 159.1. **LC-MS** *m/z* (ES+) = 513.3 $[M + 2H]^{2+}$ **YIELD:** 53%

3-{8-[-*N′*-(cyclopropylmethyl)-*N′*,*N*"-di-Boc-carbamimidamido]octyl}-1-[*N*-[8-({8-[*N′*-(cyclopropylmethyl)-*N′*,*N*"-di-Boc-carbamimidamido]octyl}[(4-methoxyphenyl)methyl]amino)octyl]-*N′*-Boc-carbamimidoyl]-3-[8-(*N′*-Boc-carbamimidamido)octyl]urea (17) To a solution of compound **16** (18.0 mg, 0.02 mmol) in *i*-PrOH (2.0 mL), Pd/C 10% (3.6 mg, 0.003 mmol) was added. The reaction mixture was subjected to 3 cycles of vacuum followed by flash of H_2, and it was stirred under a strong flux of H_2 for 5 h. The reaction mixture was diluted with CH_3OH (10.0 mL) and filtered through a plug of celite. The filtrate was evaporated under reduced pressure. The oil obtained was used for the next reaction step without any further purification. **LC-MS** *m/z* (ES+) = 487.4 $[M + 2H]^{2+}$; 325.3 $[M + 3H]^{3+}$

To a solution of the crude product in CH_3CN/ H_3OH 9/1 (1.0 mL), a solution of *N,N′*-Di-Boc-*N*- methylcyclopropyl-pyrazole-1-carboxamidine (13.0 mg, 0.04 mmol) in CH_3CN/CH_3OH 9/1 (0.5 mL) was added dropwise. After the addition of DIPEA (catalytic amount) the reaction mixture was stirred at 60 °C overnight. Then the solvent was removed under reduced

pressure and the crude product was purified trough flash column chromatography (silica gel) (DCM/CH_3OH 9/1), affording the product as a yellowish oil. ^{1}**H NMR** ($CDCl_3$, 400 MHz): δ 0.18-0.23 (m, 4H); 0.41-0.48 (m, 4H); 1.00-1.10 (m, 2H); 1.18-1.38 (m, 32H); 1.40-1.52 (m, 54H); 1.54-1.67 (m, 16H) 2.31-2.40 (m, 2H); 2.41-2.50 (m, 2H); 3.12 (t, *J* = 7.2 Hz, 4H); 3.17-3.31 (m, 8H); 3.33-3.42 (m, 2H); 3.49-3.58 (m, 4H); 3.77 (s, 3H); 6.82 (t, *J* = 8.0 Hz, 3H); 7.23 (t, *J* = 8.0 Hz, 2H); 7.90 (br, 1H). ^{13}**C NMR** ($CDCl_3$, 100 MHz): δ 3.4; 8.3; 26.7; 27.4; 28.4; 29.3; 30.3; 41.5; 42.0; 42.2; 49.8; 50.3; 55.8; 56.9; 65.0; 79.7; 82.5; 54.6; 114.0; 130.9; 132.5; 149.1; 153.7; 154.3; 155.4; 155.9; 158.1; 158.5; 159.2. **LC-MS** *m/z* (ES+) = 783.5 $[M + 2H]^{2+}$; 522.8 $[M + 3H]^{3+}$ **YIELD:** 28% (over the two reaction steps)

3-{8-[*N'*-(cyclopropylmethyl)-*N'*,*N''*-di-Boc-carbamimidamido]octyl}-1-[*N*-[8-({8-[*N'*-(cyclopropylmethyl)-*N'*,*N''*-Di-Boc-carbamimidamido]octyl}amino)octyl]-*N'*-Boc-carbamimidoyl]-3-[8-(*N'*-Boc-carbamimidamido)octyl]urea (18) Compound **17** (30.0 mg, 0.02 mmol) was dissolved in CH_3CN (0.5 mL) and a solution of cerium ammonium nitrate (6.5 mg, 0.04 mmol) in H_2O (0.1 mL) was added dropwise. The reaction mixture was stirred at room temperature overnight. Then $NaHCO_3$ s.s. (3.0 mL) and AcOEt (3.0 mL) were added ant the mixture was stirred for 10 min. The aqueous phase was extracted three times with AcOEt (3.0 mL), washed with Brine (10.0 mL), dried over Na_2SO_4 and evaporated under reduced pressure. The crude product was purified by flash column chromatography (silica gel) (DCM/CH_3OH 98/2), affording the product as a pale-yellow oil. ^{1}**H NMR** (CD_3OD, 400 MHz): δ 0.20-0.22 (m, 4H); 0.42-0.44 (m, 4H); 0.80-1-18 (m, 2H); 1.17-1.39 (m, 32H); 1.42-1.55 (m, 54H); 1.57-1.65 (m, 16H); 2.80-2.89 (m, 4H); 3.20-3.38 (m, 8H); 3.32-3.41 (m, 4H); 3.50-3.57 (m, 4H); 7.96 (br, 1H); 8.26 (br, 1H); 11.50 (br, 1H); 12.30 (br, 1H). ^{13}**C NMR** (CD_3OD, 100 MHz): δ 3.4; 8.3; 26.6; 26.8; 28.4; 29.3; 30.3; 30.6; 44.5; 42.3; 49.8; 49.9; 79.7; 82.6; 84.6; 149.1; 153.7; 154.3; 155.4; 155.8; 158.0; 158.6. **LC-MS** *m/z* (ES+) = 728.0 $[M + 2H]^{2+}$; 485.6 $[M + 3H]^{3+}$ **YIELD:** 38%

1,3-bis({8-[*N'*-(cyclopropylmethyl)-*N'*,*N''*-di-Boc-carbamimidamido]octyl})-3-(8-{[[({8-[*N'*-(cyclopropylmethyl)-*N'*,*N''*-di-Boc-carbamimidamido]octyl}[8-(*N'*-Boc-carbamimidamido)octyl]carbamoyl)amino](*N*-Boc-imino)methyl]amino}octyl)-1-{8-[*N'*,*N''*-di-Boc-carbamimidamido]octyl}urea (19) To a solution of compound **19** (22.0 mg, 0.015 mmol) in dry DCM (1.0 mL), a solution of **11** (26.0 mg, 0.03 mmol) in dry DCM (1.0 mL) was added. Then DIPEA (catalytic amount) and sodium iodide (catalytic amount) were added. The reaction mixture was stirred at reflux overnight. After cooling, the mixture was treated with $NaHCO_3$ s.s. (5.0 mL) and extracted several times with DCM (5.0 mL). The combined organic layers were washed with Brine (15.0 mL), dried over Na_2SO_4 and evaporated under reduced pressure. The crude product was purified by flash column chromatography (silica gel) (Hexane/AcOEt 3/2), affording the prooduct as a yellowish oil. ^{1}**H NMR** ($CDCl_3$, 400 MHz): δ 0.18-0.24 (m, 4H); 0.41-0.48 (m, 4H); 1.00-1.10 (m, 1H); 1.21-1.38 (m, 48H); 1.42-1.56 (m, 63H); 1.58-1.70 (m, 24H); 3.00-3.09 (m, 6H); 3.22-3.26 (m, 4H); 3.27-3.35 (m, 8H); 3.35-3.37 (m, 6H); 3.38-3.40 (m, 6H); 3.50-3.57 (m, 6H); 7.95 (br; 1H); 8.26 (br, 2H); 11.47 (br, 1H); 12.32 (br, 1H). ^{13}**C NMR** ($CDCl_3$, 100 MHz): δ 3.4; 8.4; 26.6; 28.4; 29.3; 158.6; 160.4; 164.5. **LC-MS** *m/z* (ES+) = 1141.0 $[M + 2H]^{2+}$; 761.2 $[M + 3H]^{3+}$; 571.1 $[M + 4H]^{4+}$ **YIELD:** 20%

***N*-(8-aminooctyl)-*N'*-(cyclopropylmethyl)-*N'*,*N''*-di-Boc-guanidine (20)** To a solution of 1,8-Diaminooctane (586.0 mg, 4.07 mmol) in CH_3CN/CH_3OH 9/1 (10.0 mL), a solution of *N,N'*-Di-Boc-*N*- methylcyclopropyl-pyrazole-1-carboxamidine (370.0 mg, 1.02 mmol) in CH_3CN/CH_3OH 9/1 (10.0 mL) was added. DIPEA (0.3 mL, 2.03 mmol) was added to the reaction mixture and it was stirred at 50 °C overnight. Then solvent was evaporated and the crude product was purified by flash column chromatography (silica gel) (CH_3CN/CH_3OH/TEA 8/2/2), affording the product as a yellow oil. ^{1}**H NMR** ($CDCl_3$, 400 MHz): δ 0.22-0.27 (m, 2H); 0.42-0.48 (m, 2H); 1.01-1.10 (m, 1H); 1.29-1.40 (m, 8H); 1.46 (s, 9H); 1.49 (s, 9H); 1.56-1.67 (m, 2H); 1.68-1.75 (m, 2H); 2.68 (t, *J* = 7.0 Hz, 2H); 3.32 (t, *J* = 6.8 Hz, 2H); 3.50-3.59 (m, 2H); 8.23 (br, 1H); 11.50 (br, 1H). ^{13}**C NMR** ($CDCl_3$, 100 MHz): δ 3.4; 8.4; 26.7; 27.0; 28.4; 29.3; 30.1; 30.3; 30.5; 41.9; 49.9; 50.0; 50.38; 79.9; 84.6; 153.7; 158.0; 160.4. **LC-MS** *m/z* (ES+) = 441.3 $[M + H]^{+}$ **YIELD:** 92%

***N*-{8-[(8-azidooctyl)amino]octyl}-*N'*-(cyclopropylmethyl)-*N'*,*N''*-di-Boc-guanidine (21)** To cesium hydroxide monohydrate (78.0 mg, 0.47 mmol) and molecular sieves (200.0 mg) dry DMF (2.5 mL) was added under nitrogen atmosphere and the mixture was stirred for 10 min. A solution of **20** (205.0 mg, 0.47 mmol) in dry DMF (2.5 mL) was added and the mixture was stirred for further 30 min. Then **6** (87.0 mg, 0.37 mmol) was added and the reaction mixture was stirred at room temperature overnight. The mixture was diluted with AcOEt (5.0 mL), filtered from the solid, washed and concentrated under vacuum. The residue was treated with NaOH 1N (10.0 mL) and extracted three times with AcOEt (10.0 mL). The combined organic phases were washed with H_2O (30.0 mL), LiCl 5% (30.0 mL) and Brine (30.0 mL). The crude product was purified by flash column chromatography (silica gel) (DCM/CH_3OH 8/2), affording the product as a colourless oil. **^{1}H NMR** ($CDCl_3$, 400 MHz): δ 0.24 (d, *J* = 4.8 Hz, 2H); 0.45 (d, *J* = 7.6 Hz, 2H); 1.00-1.10 (m, 1H); 1.22-1.28 (m, 24H); 1.44 (s, 18H); 2.62 (t, *J* = 7.0 Hz, 4H); 3.17 (t, *J* = 6.8 Hz, 2H); 3.18-3.25 (m, 2H); 3.45 (q, *J* = 5.6 Hz, 2H); 8.23 (br, 1H); 11.50 (br, 1H). **^{13}C NMR** ($CDCl_3$, 100 MHz): δ 3.4; 8.3; 26.; 27.0; 28.4; 29.3; 30.1; 30.3; 30.5; 41.9; 49.9; 50.0; 50.4; 79.9; 84.6; 153.7; 158.0; 160.4. **LCMS** *m/z* (ES+) = 594.3 $[M + H]^+$ **YIELD:** 43%

***N'*-(cyclopropylmethyl)-*N*-(8-{[8-(*N'*,*N"*-di-Boc-carbamimidamido)octyl](Fmoc)amino}octyl)-*N'*,*N"*-di-Boc-guanidine (22)** To a solution of **10** (100.0 mg, 0.12 mmol) in dry DCM (10.0 mL), TEA (20.0 µL, 0.15 mmol) and fluorenylmethyloxycarbonyl chloride (38.0 mg, 0.15 mmol) were added at 0 °C. The reaction mixture was stirred at 0 °C for 30 min. Then the mixture was treated with H_2O (20.0 mL) and extracted several times with DCM (20 mL). The combined organic layers were washed with H_2O (20.0 mL) and Brine (20.0 mL), dried over Na_2SO_4 and evaporated under reduced pressure. The crude product was purified by flash column chromatography (silica gel) (Hexane/AcOEt 2/1), affording the product as a yellowish oil. **^{1}H NMR** ($CDCl_3$, 400 MHz): δ 0.19-0.23 (m, 2H); 0.39-0.43 (m, 2H,); 1.00-1.17 (m, 1H); 1.20-1.33 (m, 16H); 1.42-1.60 (m, 36H); 1.62-1.70 (m, 8H); 2.90-3.01 (m, 2H); 3.10-3.19 (m, 2H); 3.23-3.31 (m, 2H); 3.32-3.43 (m, 2H); 3.49-3.60 (m, 2H); 4.18 (t, *J* = 6.0 Hz, 1H); 4.46 (d, *J* = 6.0 Hz, 2H); 7.25 (t, *J* = 4.4 Hz, 2H) 7.35 (t, *J* = 7.6 Hz, 2H) 7.54 (d, *J* = 7.2 Hz, 2H) 7.72 (d, *J* = 7.6 Hz, 2H); 8.26 (br, 1H); 11.48 (br, 1H). **^{13}C NMR** ($CDCl_3$, 100 MHz): δ 3.5; 10.5; 26.7; 26.9; 28.0; 28.2; 28.2; 28.9; 29.2; 40.9; 43.8; 47.0; 47.4; 52.0; 66.4; 79.1; 82.9; 119.8; 124.7; 126.9; 127.5; 141.3; 144.1; 153.2; 156.0; 163.6. **LC-MS** *m/z* (ES+) = 516.5 $[M + 2H]^{2+}$; 344.7 $[M + 3H]^{3+}$ **YIELD:** 74%

1-(8-azidooctyl)-1-{8-[*N'*-(cyclopropylmethyl)-*N'*,*N''*-di-Boc-carbamimidamido]octyl}-3-{*N*-[8-({8-[*N'*-(cyclopropylmethyl)-*N'*,*N''*-di-Boc-carbamimidamido]octyl}amino)octyl]-*N'*-Boc-carbamimidoyl}urea (23) To a solution of **22** (55.0 mg, 0.05 mmol) in THF (2.0 mL) a solution of **21** (32.0 mg, 0.05 mmol) in THF (3.0 mL) and a TEA (8.0 µL, 0.05 mmol) were added and the reaction mixture was stirred at reflux for 10 h. After cooling, the mixture was treated with $NaHCO_3$ s.s. (15.0 mL) and extracted several times with AcOEt (15.0 mL). The combined organic layers were washed with Brine (50.0 mL), dried over Na_2SO_4 and evaporated under reduced pressure. The crude product was purified with by flash column chromatography (silica gel) (Hexane/AcOEt 2/1), affording the product as a yellowish oil. **^{1}H NMR** ($CDCl_3$, 400 MHz): δ 0.18-0.22 (m, 4H); 0.39-0.43 (m, 4H); 1.00-1.12 (m, 1H); 1.20-1.29 (m, 32H); 1.40-1.52 (m, 45H); 1.53-1.64 (m, 16H); 2.93-3.01 (m, 2H); 3.10-3.19 (m, 4H); 3.20-3.25 (m, 8H); 3.26-3.31 (m, 2H); 3.44-3.60 (m, 4H); 4.18 (t, *J* = 6.0 Hz, 1H); 4.45 (d, *J* = 5.6 Hz, 2H); 5.15 (br; 1H); 7.26 (t, *J* = 10.0 Hz, 2H) 7.35 (t, *J* = 7.2 Hz, 2H); 7.54 (d, *J* = 8.0 Hz, 2H); 7.72 (d, *J* = 8.0 Hz, 2H); 7.94 (br, 1H); 12.14 (br, 1H). **^{13}C NMR** ($CDCl_3$, 100 MHz): δ 3.4; 8.4 11.2; 26.8; 27.0; 28.5; 28.9; 29.3; 30.1; 30.3; 41.6; 42.3; 44.7; 47.0; 49.8; 50.4; 51.1; 67.6; 79.8; 82.5; 120.5; 125.2; 126.2; 126.8; 142.7; 143.6; 149.2; 151.8; 154.2; 155.9; 158.0; 158.6; 159.8. **LC-MS** *m/z* (ES+) = 776.0 $[M + 2H]^{2+}$; 518.0 $[M + 3H]^{3+}$ **YIELD:** 48%

3-{8-[({[(8-azidooctyl)({8-[*N'*-(cyclopropylmethyl)-*N'*,*N''*-di-Boc-carbamimidamido]octyl})carbamoyl]amino}(Boc-imino)methyl)amino]octyl}-3-{8-[*N'*-(cyclopropylmethyl)-*N'*,*N''*-di-Boc-carbamimidamido]octyl}-1-{N-[8-({8-[N'-(cyclopropylmethyl)-*N'*,*N''*-di-Boc-carbamimidamido]octyl}(Fmoc)amino)octyl]-*N'*-Boc-carbamimidoyl}urea (24) Compound **23** (40.0 mg, 0.03 mmol) was dissolved in a 20% solution of Piperidine in DMF (3.04 mmol, 1.5 mL). The reaction

mixture was stirred at room temperature for 1 h. Then the mixture was treated with H_2O (5.0 mL) and extracted several times with AcOEt (5.0 mL). The combined organic layers were washed with Brine (15.0 mL), dried over Na_2SO_4 and evaporated under reduced pressure. No further purification followed. The crude product was used for the next reaction step. **LC-MS** *m/z* (ES+) = 665.5 $[M + 2H]^{2+}$

To the crude product, a solution of **22** in THF (4.0 mL), then TEA (4.0 µL, 0.03 mmol) was added. The reaction mixture was stirred at reflux overnight. After cooling, the mixture was treated with H_2O (5.0 mL) and extracted several times with AcOEt (5.0 mL). The combined organic layers were washed with Brine (15.0 mL), dried over Na_2SO_4 and evaporated under reduced pressure. The crude product was purified by flash column chromatography (silica gel) (Hexane/AcOEt 2/1), affording the product as a yellowish oil. **^{1}H NMR** ($CDCl_3$, 400 MHz): δ 0.18-0.24 (m, 6H); 0.41-0.45 (m, 6H); 1.00-1.10 (m, 3H); 1.25-1.35 (m, 72H); 1.40-1.49 (m, 72H); 2.98-3.02 (m, 2H); 3.12-3.18 (m, 4H); 3.24-3.26 (m, 8H); 3.27-3.29 (m, 8H); 3.37-3.41 (m, 2H); 3.48-3.52 (m, 6H); 4.20 (t, *J* = 5.7 Hz, 1H) 4.47 (d, *J* = 6.0 Hz, 2H); 5.15 (br; 1H); 7.28 (t, *J* = 7.4 Hz, 2H) 7.37 (t, *J* = 7.4 Hz, 2H); 7.56 (d, *J* = 7.4 Hz, 2H); 7.74 (d, *J* = 7.4 H, 2Hz); 7.97 (br, 1H); 12.15 (br, 1H). **^{13}C NMR** ($CDCl_3$, 100 MHz): δ 3.4; 8.3; 11.2; 26.6; 26.8; 28.4; 29.3; 30.2; 30.3; 45.5; 42.2; 44.7; 47.0; 49.9; 50.9; 51.2; 67.7; 79.8; 82.5; 120.6; 125.2; 126.2; 126.8; 142.7; 143.6; 149.1; 151.9; 154.3; 155.8; 158.0; 158.5; 159.8. **LC-MS** *m/z* (ES+) = 763.0 $[M + 3H]^{3+}$; 572.7 $[M + 4H]^{4+}$ **YIELD:** 29% (over the two reaction steps)

1-{8-[*N′*-(cyclopropylmethyl)-*N′*,*N′′*-di-Boc-carbamimidamido]octyl}-3-{*N*-[8-({8-[*N′*-(cyclopropylmethyl)-*N′*,*N′′*-di-Boc-carbamimidamido]octyl}({[*N*-[8-({8-[*N′*-(cyclopropylmethyl)-*N′*,*N′′*-di-Boc-carbamimidamido]octyl}amino)octyl]-*N′*-Boc-carbamimidoyl]carbamoyl})amino)octyl]-*N′*-Boc-carbamimidoyl}-1-{8-[*N′*,*N′′*-di-Boc-carbamimidamido]octyl}urea (25)

To a solution of **24** (20.0 mg, 0.01 mmol) in *i*-PrOH (2.0 mL), Pd/C 10% (2.0 mg, 0.001 mmol) was added. The reaction mixture was subjected to 3 cycles of vacuum followed by flash of H_2, and it was stirred under a strong flux of H_2 for 1.5 h. The reaction mixture was diluted with CH_3OH (10.0 mL) and filtered through a plug of celite. The filtrate was evaporated under reduced pressure. The oil obtained was used for the next reaction step without any further purification. **LC-MS** *m/z* (ES+) = 754.5 $[M + 3H]^{3+}$; 766.0 $[M + 4H]^{4+}$

To a solution of the crude product in THF (1.0 mL), *N,N′*-Di-Boc-1*H*-pyrazole-1-carboxamidine (5.4 mg, 0.015 mmol) and DIPEA (catalytic amount) were added. The reaction mixture was stirred at room temperature overnight. Then the mixture was treated with $NaHCO_3$ s.s. (5.0 mL) and extracted several times with AcOEt (5.0 mL). The combined organic layers were washed with Brine (15.0 mL), dried over Na_2SO_4 and evaporated under reduced pressure. No further purification followed, the yellowish oil obtained was used for the next reaction step. **LC-MS** *m/z* (ES+) = 835.7 $[M + 3H]^{3+}$

The crude product was dissolved in a 20% solution of Piperidine in DMF (2.02 mmol, 1.0 mL). The reaction mixture was stirred at room temperature for 5 h. Then the mixture was treated with $NaHCO_3$ s.s. (6.0 mL) and extracted several times with AcOEt (5.0 mL). The combined organic layers were washed with Brine (15.0 mL), dried over Na_2SO_4 and evaporated under reduced pressure. The crude product was purified by flash column chromatography (silica gel) (DCM/CH_3OH 95/5), affording the product as a yellowish oil. **^{1}H NMR** ($CDCl_3$, 400 MHz): δ 0.18-0.23 (m, 6H); 0.41-0.47 (m, 6H); 1.00-1.10 (m, 3H); 1.22-1.40 (m, 48H); 1.42-1.51 (m, 90H); 1.55-1.70 (m, 24H); 2.79-2.87 (m, 4H); 3.21-3.30 (m, 14H); 3.33-3.43 (m, 6H); 3.50-3.58 (m, 6H); 7.98 (br; 2H); 8.28 (br, 1H); 11.49 (br, 1H); 12.16 (br, 1H). **^{13}C NMR** ($CDCl_3$, 100 MHz): δ 3.4; 8.4; 11.2; 26.7; 27.0; 28.5; 29.3; 30.3; 30.5; 41.5; 42.2; 44.8; 49.8; 79.8; 82.5; 149.1; 153.8; 154.2; 158.1; 158.0; 158.6; 159.8. **LC-MS** *m/z* (ES+) = 761.2 $[M + 3H]^{3+}$ **YIELD:** 20% (over the three reaction steps)

Proposed mechanism of urea moiety formation in compounds 2 and 4.

Both the symmetric oligomers (dimer **2** and trimer **4**) are characterized by an urea moiety. In order to explain the source of this carbonyl group that links the central amine of the monomers (compound **1**), two possible mechanisms are reported.

According to the first hypothesis (**Figure S8**), a reaction between the central amine of compound **1** and atmospheric carbon dioxide occurs: the nucleophilic addition is followed by a proton transfer furnishing the corresponding carbamic acid that reacts with another molecule of compound **1**. The dehydration of the obtained adduct gave the urea moiety. This transformation has been exhaustively described [S1] as a convenient strategy to capture waste carbon dioxide through the linkage with amine functional adsorbents.

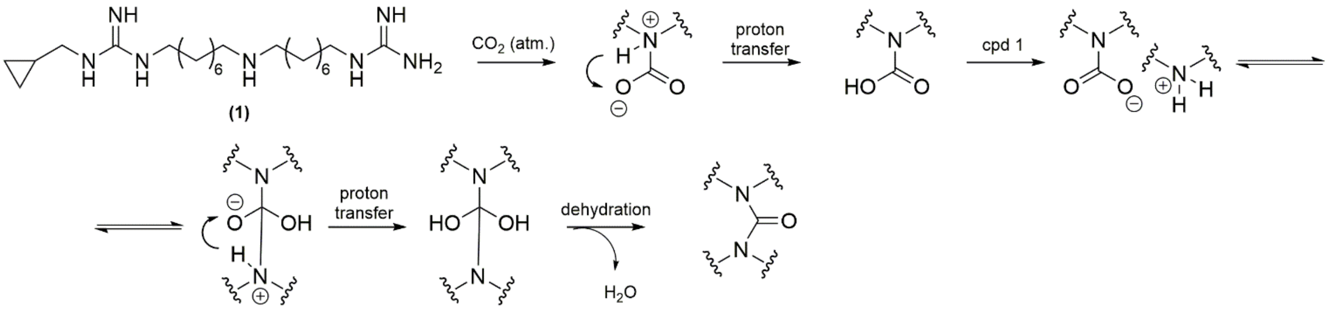

Figure S8. First putative mechanism of urea moiety formation involving atmospheric carbon dioxide.

The second proposed mechanism (**Figure S9**) involves a macrocyclic derivative, whose formation could occur during the monomer synthesis, particularly with the guanylation reaction, as already reported in our previous work.[S2] The concomitant presence of the monomer (compound **1**) and this macrocyclic byproduct in the storage solution allowed the nucleophilic addition of the central amine of compound **1** to the carbonyl group of the amidinourea macrocycle. The following ring opening furnished the urea moiety.

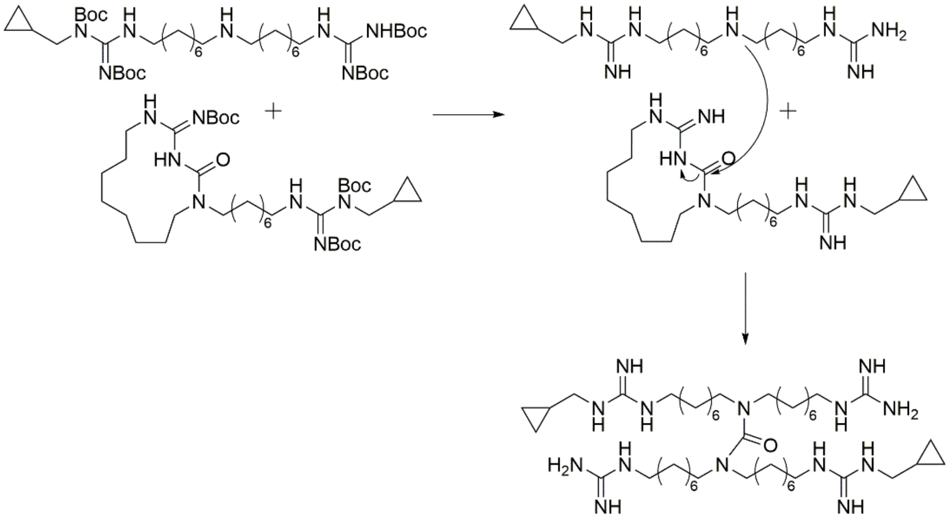

Figure S9. Second putative mechanism of urea moiety formation involving a macrocyclic byproduct.

Kill curve assay of compound 2. The bactericidal activity of compound **2** was investigated through a kill curve assay. It was performed on *S. aureus* ATCC 25923 (starting inoculum, 2.5 x 10^8 CFU/mL) in the presence of 20 µg/mL of compound **2** (10 × MIC). Residual viable count was determined after 1, 2, 3, 4 and 24 hours of exposure by plating serial 10-fold dilutions of the cultures on Mueller-Hinton agar plates, subsequently incubated for 18-24 hours at 35 °C prior to colony counting. Vancomycin (10 µg/ml, 10 × MIC) was used as a comparator.

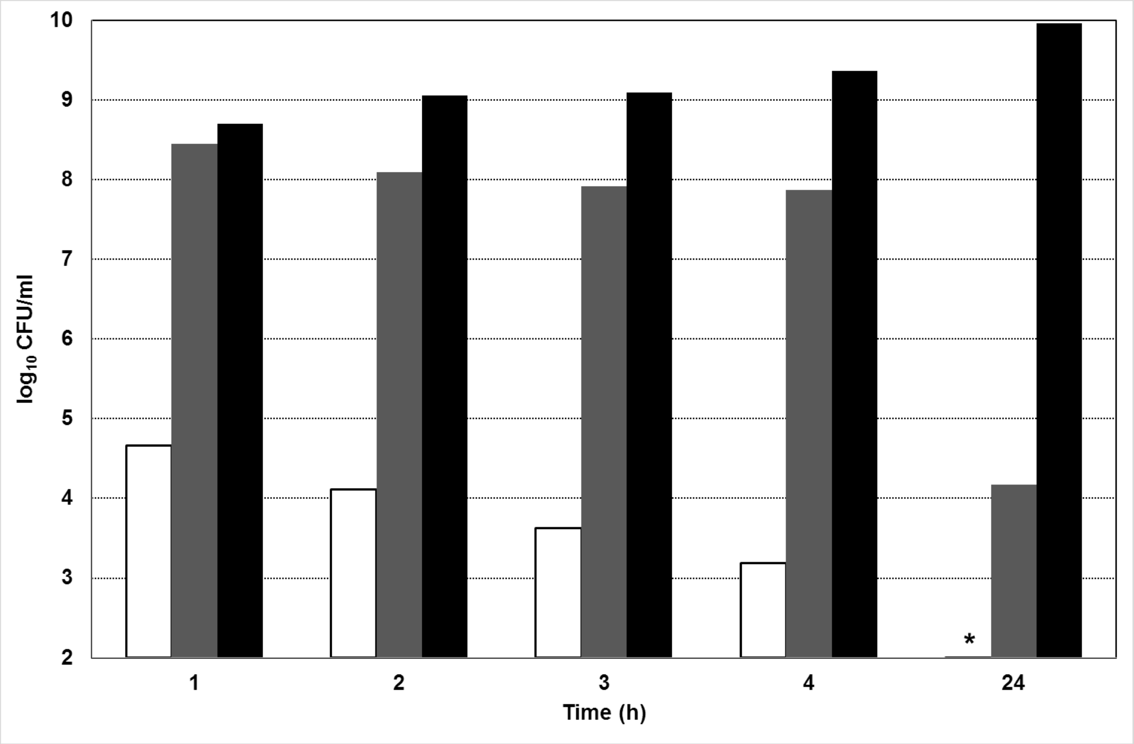

Figure S10. Kill curve of compound **2** on *S. aureus* ATCC 25923.
Viable count of *S. aureus* ATCC 25923 exposed to 10 × MIC of compound **2** (white bars) and vancomycin (grey bars). Growth control is shown as black bars.
*: viable count ≤ 10 CFU/mL.

Supplementary Table S1. Molecular formula strings of compounds **1-25**

Cpd	SMILE
**1**	NC(NCCCCCCCCNCCCCCCCCNC(NCC1CC1)=N)=N
**2**	NC(NCCCCCCCCN(C(N(CCCCCCCCNC(N)=N)CCCCCCCCNC(NCC1CC1)=N)=O)CCCCCCCCNC(NCC2CC2)=N)=N
**3**	NC(NCCCCCCCCN(C(NC(NCCCCCCCCNCCCCCCCCNC(NCC1CC1)=N)=N)=O)CCCCCCCCNC(NCC2CC2)=N)=N
**4**	NC(NCCCCCCCCN(C(NC(NCCCCCCCCN(C(N(CCCCCCCCNC(NCC1CC1)=N)CCCCCCCCNC(N)=N)=O)CCCCCCCCNC(NCC2CC2)=N)=N)=O)CCCCCCCCNC(NCC3CC3)=N)=N
**5**	NC(NCCCCCCCCN(C(NC(NCCCCCCCCN(C(NC(NCCCCCCCCNCCCCCCCCNC(NCC1CC1)=N)=N)=O)CCCCCCCCNC(NCC2CC2)=N)=N)=O)CCCCCCCCNC(NCC3CC3)=N)=N
**6**	BrCCCCCCCCN=[N+]=[N-]
**7**	NCCCCCCCCNC(NC(OC(C)(C)C)=O)=NC(OC(C)(C)C)=O
**8**	CC(OC(NC(NCCCCCCCCNCCCCCCCCN=[N+]=[N-])=NC(OC(C)(C)C)=O)=O)(C)C
**9**	NCCCCCCCCNCCCCCCCCNC(NC(OC(C)(C)C)=O)=NC(OC(C)(C)C)=O
**10**	CC(OC(NC(NCCCCCCCCNCCCCCCCCNC(N(CC1CC1)C(OC(C)(C)C)=O)=NC(OC(C)(C)C)=O)=NC(OC(C)(C)C)=O)=O)(C)C
**11**	O=C(Cl)N(CCCCCCCCNC(N(CC1CC1)C(OC(C)(C)C)=O)=NC(OC(C)(C)C)=O)CCCCCCCCNC(NC(OC(C)(C)C)=O)=NC(OC(C)(C)C)=O
**12**	O=C(N(CCCCCCCCNC(NC(OC(C)(C)C)=O)=NC(OC(C)(C)C)=O)CCCCCCCCNC(N(CC1CC1)C(OC(C)(C)C)=O)=NC(OC(C)(C)C)=O)N(CCCCCCCCNC(N(CC2CC2)C(OC(C)(C)C)=O)=NC(OC(C)(C)C)=O)CCCCCCCCNC(NC(OC(C)(C)C)=O)=NC(OC(C)(C)C)=O
**13**	COC(C=C1)=CC=C1CNCCCCCCCCNC(NC(OC(C)(C)C)=O)=NC(OC(C)(C)C)=O
**14**	COC(C=C1)=CC=C1CN(CCCCCCCCNC(NC(OC(C)(C)C)=O)=NC(OC(C)(C)C)=O)CCCCCCCCN=[N+]=[N-]
**15**	N=C(NC(OC(C)(C)C)=O)NCCCCCCCCNCCCCCCCCN=[N+]=[N-]
**16**	N=C(NC(OC(C)(C)C)=O)NCCCCCCCCN(C(NC(NCCCCCCCCN(CC1=CC=C(OC)C=C1)CCCCCCCCN=[N+]=[N-])=NC(OC(C)(C)C)=O)=O)CCCCCCCCN=[N+]=[N-]
**17**	N=C(NC(OC(C)(C)C)=O)NCCCCCCCCN(C(NC(NCCCCCCCCN(CC1=CC=C(OC)C=C1)CCCCCCCCNC(N(CC2CC2)C(OC(C)(C)C)=O)=NC(OC(C)(C)C)=O)=NC(OC(C)(C)C)=O)=O)CCCCCCCCNC(N(CC3CC3)C(OC(C)(C)C)=O)=NC(OC(C)(C)C)=O
**18**	N=C(NC(OC(C)(C)C)=O)NCCCCCCCCN(C(NC(NCCCCCCCCNCCCCCCCCNC(N(CC1CC1)C(OC(C)(C)C)=O)=NC(OC(C)(C)C)=O)=NC(OC(C)(C)C)=O)=O)CCCCCCCCNC(N(CC2CC2)C(OC(C)(C)C)=O)=NC(OC(C)(C)C)=O
**19**	N=C(NC(OC(C)(C)C)=O)NCCCCCCCCN(C(NC(NCCCCCCCCN(C(N(CCCCCCCCNC(N(CC1CC1)C(OC(C)(C)C)=O)=NC(OC(C)(C)C)=O)CCCCCCCCNC(NC(OC(C)(C)C)=O)=NC(OC(C)(C)C)=O)=O)CCCCCCCCNC(N(CC2CC2)C(OC(C)(C)C)=O)=NC(OC(C)(C)C)=O)=NC(OC(C)(C)C)=O)=O)CCCCCCCCNC(N(CC3CC3)C(OC(C)(C)C)=O)=NC(OC(C)(C)C)=O
**20**	NCCCCCCCCNC(N(C(OC(C)(C)C)=O)CC1CC1)=NC(OC(C)(C)C)=O
**21**	[N-]=[N+]=NCCCCCCCCNCCCCCCCCNC(N(C(OC(C)(C)C)=O)CC1CC1)=NC(OC(C)(C)C)=O
**22**	CC(OC(NC(NCCCCCCCCN(C(OCC1C(C=CC=C2)=C2C3=C1C=CC=C3)=O)CCCCCCCCNC(N(C(OC(C)(C)C)=O)CC4CC4)=NC(OC(C)(C)C)=O)=NC(OC(C)(C)C)=O)=O)(C)C

**23**	O=C(NC(NCCCCCCCCN(C(OCC1C(C=CC=C2)=C2C3=C1C=CC=C3)=O)CCCCCCCCNC(N(C(OC(C)(C)C)=O)CC4CC4)=NC(OC(C)(C)C)=O)=NC(OC(C)(C)C)=O)N(CCCCCCCCN=[N+]=[N-])CCCCCCCCNC(N(C(OC(C)(C)C)=O)CC5CC5)=NC(OC(C)(C)C)=O
**24**	O=C(NC(NCCCCCCCCN(C(NC(NCCCCCCCCN(C(OCC1C(C=CC=C2)=C2C3=C1C=CC=C3)=O)CCCCCCCCNC(N(C(OC(C)(C)C)=O)CC4CC4)=NC(OC(C)(C)C)=O)=NC(OC(C)(C)C)=O)=O)CCCCCCCCNC(N(C(OC(C)(C)C)=O)CC5CC5)=NC(OC(C)(C)C)=O)=NC(OC(C)(C)C)=O)N(CCCCCCCCN=[N+]=[N-])CCCCCCCCNC(N(C(OC(C)(C)C)=O)CC6CC6)=NC(OC(C)(C)C)=O
**25**	O=C(NC(NCCCCCCCCN(C(NC(NCCCCCCCCNCCCCCCCCNC(N(C(OC(C)(C)C)=O)CC1CC1)=NC(OC(C)(C)C)=O)=NC(OC(C)(C)C)=O)=O)CCCCCCCCNC(N(C(OC(C)(C)C)=O)CC2CC2)=NC(OC(C)(C)C)=O)=NC(OC(C)(C)C)=O)N(CCCCCCCCNC(NC(OC(C)(C)C)=O)=NC(OC(C)(C)C)=O)CCCCCCCCNC(N(C(OC(C)(C)C)=O)CC3CC3)=NC(OC(C)(C)C)=O

Supplementary References:

S1. Li, K. *et al.* Research on Urea Linkages Formation of Amine Functional Adsorbents During CO 2 Capture Process : Two Key Factors Analysis , Temperature and Moisture. (2016). doi:10.1021/acs.jpcc.6b08788

S2. Castagnolo, D., Raffi, F., Giorgi, G. & Botta, M. Macrocyclization of di-Boc-guanidino-alkylamines related to guazatine components: Discovery and synthesis of innovative macrocyclic amidinoureas. *European J. Org. Chem.* 334–337 (2009). doi:10.1002/ejoc.200801109
